# Thyroid hormone regulates glutamine metabolism and anaplerotic fluxes by inducing mitochondrial glutamate aminotransferase GPT2

**DOI:** 10.1016/j.celrep.2022.110409

**Published:** 2022-02-22

**Authors:** Annunziata Gaetana Cicatiello, Serena Sagliocchi, Annarita Nappi, Emery Di Cicco, Caterina Miro, Melania Murolo, Mariano Stornaiuolo, Monica Dentice

**Affiliations:** 1 Department of Clinical Medicine and Surgery, University of Naples “Federico II”, Naples, NA 80138 Italy; 2 Department of Pharmacy, University of Naples “Federico II”, Naples, NA 80138 Italy; 3 CEINGE–Biotecnologie Avanzate Scarl, Naples, NA 80131, Italy

**Keywords:** thyroid hormone, type 2 deiodinase, GPT2, glutamine metabolism, skeletal muscle

## Abstract

Thyroid hormones (THs) are key metabolic regulators coordinating short- and long-term energy needs. In skeletal muscle, THs modulate energy metabolism in pathophysiological conditions. Indeed, hypo- and hyperthyroidism are leading causes of muscle weakness and strength; however, the metabolic pathways underlying these effects are still poorly understood. Using molecular, biochemical, and isotope-tracing approaches combined with mass spectrometry and denervation experiments, we find that THs regulate glutamine metabolism and anaplerotic fluxes by up-regulating the glutamate pyruvate transaminase 2 (*GPT2*) gene. In humans, *GPT2* autosomal recessive mutations cause a neurological syndrome characterized by intellectual disability, microcephaly, and progressive motor symptoms. Here, we demonstrate a role of the TH/GPT2 axis in skeletal muscle in which it regulates muscle weight and fiber diameter in resting and atrophic conditions and results in protection from muscle loss during atrophy. These results describe an anabolic route by which THs rewire glutamine metabolism toward the maintenance of muscle mass.

## Highlights

• THs induce the expression of the mitochondrial *GPT2* gene in skeletal muscle
• The GPT2 up-regulation by THs enhances anaplerotic cycles and α-KG production
• GPT2 is reduced during muscle atrophy and is reactivated by THs treatment
• GPT2 KO^+/–^ mice undergo muscle loss that is partially attenuated by THs

## Graphical abstract

**Figure:**
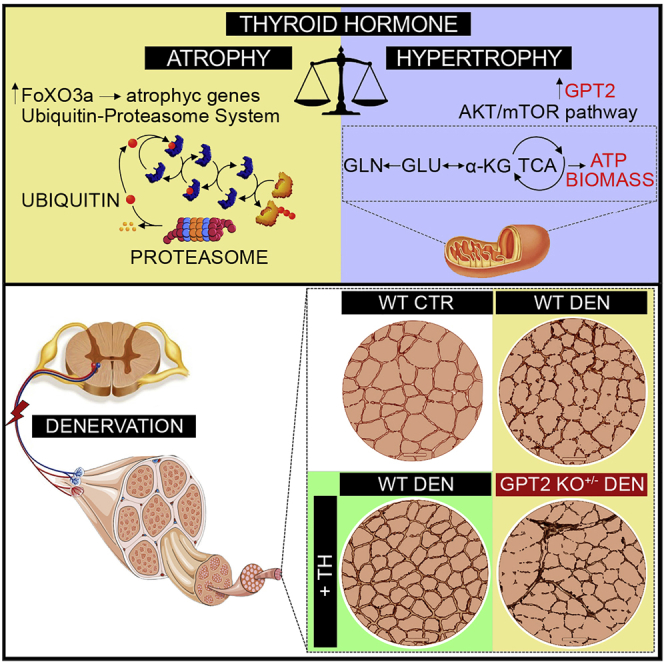

## Teaser

Using molecular, biochemical, and isotope-tracing approaches combined with mass spectrometry and denervation experiments, Cicatiello et al. demonstrate that thyroid hormones (THs) drive a rewiring of glutamine metabolism in skeletal muscle, resulting in protection from atrophy, and identify GPT2 as a critical mediator of the TH-dependent anabolic function.

## Introduction

Thyroid hormones (THs; T3 and T4) are pleiotropic agents that influence the growth, differentiation, and metabolism of virtually every cell in the body. The intracellular concentration of THs is regulated by the central hypothalamic-pituitary axis and by peripheral control of TH activation. The latter occurs via at least three steps: (1) regulation of T3 and T4 uptake into the cell by several specific transporters; (2) intracellular activation/inactivation by the deiodinases; and (3) binding of T3 to nuclear receptor transcriptional factors (Gereben et al., 2008). Besides the classical genomic action, TH and TH receptors (THRs) also have non-genomic regulatory pathways by binding to intracytosolic partners (Nappi et al., 2021; Brent, 2012). Deiodination is a potent pre-receptor mechanism that governs the activation and inactivation of THs catalyzed by three selenodeiodinases (D1, D2, and D3), which catalyze the conversion of T4 to T3 (by D1 or D2) or the inactivation of T4 and T3 mediated by D3 (Luongo et al., 2019). Because of this mechanism, D2-expressing cells are characterized by increased intracellular concentrations of active T3 and by the enhanced expression of TH target genes (Marsili et al., 2011; Ambrosio et al., 2013).

THs are key regulators of many important metabolic pathways (Cicatiello et al., 2018), and TH status finely correlates with body weight and energy expenditure (Mullur et al., 2014; Iwen et al., 2013). Indeed, hyperthyroidism accelerates the basal metabolic state and the resting energy expenditure, induces weight loss, and increases lipolysis and gluconeogenesis despite increased food intake (Cicatiello et al., 2018; Salvatore et al., 2014). The potency of THs in reducing weight loss is partially due to their ability to promote futile cycles and to reduce metabolic efficiency. Indeed, THs dissipate energy by stimulating lipogenesis, lipolysis, protein synthesis and degradation, gluconeogenesis, and glycolysis in many target tissues (Yehuda-Shnaidman et al., 2014). Moreover, induction of adaptive thermogenesis by D2 in brown adipose tissue is a paradigm of the energy dissipation induced by THs (Cicatiello et al., 2018; de Jesus et al., 2001; Bianco and McAninch, 2013). Although the role of the TH in modulating metabolic efficiency has been known for over a century, its cellular mode of action on specific metabolic pathways has only partially been elucidated.

In an attempt to shed light on the biochemical pathways modulated by THs in skeletal muscle, we investigated the effects of the D2-mediated TH activation in muscle cells by using metabolic profiling and isotope-tracing approaches and found that the TH-dependent effects exerted on glutamine metabolism are mediated by the glutamine aminotransferase GPT2 (also known as ALT2), which we identify as a TH target gene in muscle cells and tissues. GPT2 was potently induced by THs, which resulted in a metabolic shift by enhancing glutamine addiction and consumption. We demonstrate that the GPT2 induced by THs drives muscle hypertrophy and prevents acute muscle loss in experimental models of denervation-induced muscle atrophy. Moreover, THs induced a slow-to-fast fiber shift that was associated with muscle protection from atrophy. When compared with amino acid supplementation, the TH treatment was more effective in protecting from muscle loss since THs induced a global change of fiber composition that largely protected against muscle loss. Finally, using a genetic mouse model of GPT2 loss of function, we confirmed that the pro-hypertrophic action of THs following denervation is dependent on the anaplerotic role of GPT2 in skeletal muscle. Overall, these data indicate a further action of THs that, beside their role in mediating muscle catabolism through the induction of FoxO3a (Dentice et al., 2014), act also major regulators of glutamine metabolism in skeletal muscle and point to a previously unappreciated effect of THs, which dictates glutamine metabolism destiny during catabolic conditions.

## Results

### TH activation regulates glutamine metabolism

**Figure 1:**
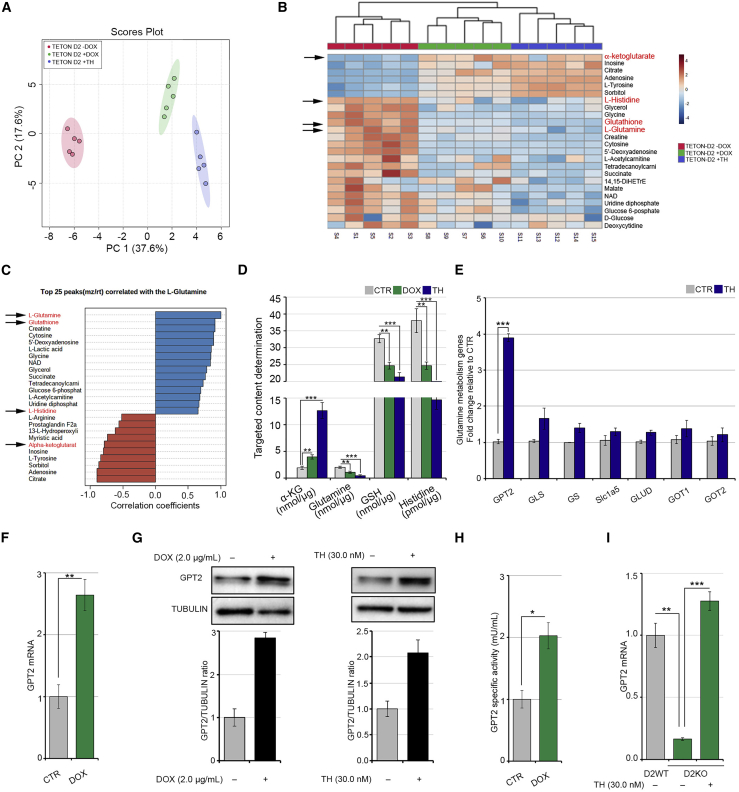
Thyroid hormone induces metabolic changes in muscle cells by enhancing glutamine metabolism (A) Principal-component analysis for metabolites profiled by GC/MS and extracted from C2C12 pTRE-D2 cells treated for 48 h with 2 μg/mL DOX, THs, and in untreated control (CTR) cells. The experiments were performed on five different biological replicates that were run in technical triplicates. (B) Heatmap analysis for metabolites profiled by GC/MS as in (A). The rows show metabolites, and the columns represent the samples, p < 0.001. (C) The top 25 peaks correlated with glutamine from the same analysis as in (A). (B and C) Black arrows indicate the key metabolites involved in glutamine metabolism. (D) Levels of α-KG, glutamine, GSH, and histidine determined by targeted GC-MS analysis in the same cells as in (A). (E) mRNA expression levels of a panel of genes involved in glutamine metabolism were measured by real-time PCR in C2C12 cells cultured with or without THs for 24 h. (F) mRNA expression levels of the *GPT2* gene in C2C12 pTRE-D2 cells cultured with or without 2 μg/mL DOX for 48 h. (G) Protein levels of GPT2 were measured by western blot analysis in C2C12 pTRE-D2 cells treated or not with 2 μg/mL DOX (left) and in C2C12 cells treated or not with THs (right) for 48 h. The histograms below show the quantification of the GPT2 protein levels versus tubulin levels. (H) Enzymatic activity of GPT2 was measured in C2C12 pTRE-D2 cells treated or not with 2 μg/mL DOX for 48 h. (I) mRNA expression levels of *GPT2* gene were measured in D2 wild-type (D2WT) muscle stem cells and in D2-knock out (D2KO) muscle stem cells treated or not with THs for 48 h. Cyclophilin-A served as an internal CTR. Normalized copies of the indicated genes in CTR cells were set as 1. Data represent the mean ± standard deviation of the mean of fifteen replicates. ^∗^p < 0.05, ^∗∗^p < 0.01, ^∗∗∗^p < 0.001.

In an attempt to identify the biochemical pathways modulated by THs in skeletal muscle cells, we metabolically profiled the D2-overexpressing and TH-treated muscle cells by gas chromatography/mass spectrometry (GC/MS). To overexpress D2, we used the pTRE-D2 C2C12 cell line in which the *Dio2* gene can be overexpressed by doxycycline (DOX) through the tetracycline regulation system (TET-ON) (Sagliocchi et al., 2019). We screened for metabolites that were altered when D2-dependent TH activation was induced by DOX treatment and when THs were added to the cells. Principal-component analysis and dendrograms confirmed that DOX- and TH-treated C2C12 cells share similar metabolic landscapes (Figures 1A, 1B, and S1A). As expected, D2 overexpression and TH treatment altered the intracellular levels of several metabolites (Figure 1C). Top candidates were confirmed and quantified using a targeted approach in independent sets of experiments (Figure 1D). Notably, glutamine was among the metabolites reduced by D2 overexpression (Figures 1B–1D). Among the different intracellular reactions in which it is involved, glutamine can be used to produce glutathione (GSH). Surprisingly, intracellular GSH was also reduced by D2 (Figures 1B–1D). On the contrary, alpha-ketoglutarate (α-KG), produced by glutamate deamination, was enhanced in both TH-treated and D2-overexpressing cells (Figures 1B–1D), which suggests that D2 can switch on anaplerotic reactions that convert glutamine into Krebs-cycle intermediates. To gain further insight into the role of THs in glutamine metabolism, we measured the expression of various genes involved in glutamine metabolism regulation. Among these, glutamate pyruvate transaminase 2 (GPT2) mRNA was significantly up-regulated (Figures 1E and S1B), while glutaminase (GLS), glutamine synthase (GS), glutamine transporter (SLC1A5), glutamate transaminase (GLUD), and glutamic oxaloacetic transaminase (GOT1 and GOT2) were unchanged or only modestly increased by THs in C2C12 cells (Figures 1E and S1C). Expression of additional glutamine transporters such as SLC6A19, SLC7A5, and SLC7A8 did not change following TH treatment (Figure S1D). Similar results were obtained using a second housekeeping gene as normalizer (Figure S1F). Together, these results suggest that GPT2 is the main effector of TH-dependent glutamine metabolism. Indeed, the molecular analysis revealed up-regulation of GPT2 expression at both mRNA and protein levels in DOX- and TH-treated cells (Figures 1E–1G and S1B). Consistently, enzymatic activity was stimulated in DOX-treated cells (Figure 1H). Notably, GPT2 mRNA expression was reduced in D2-knock out (D2KO) satellite cells and rescued by TH treatment, thereby confirming TH-dependent GPT2 induction (Figure 1I). Moreover, we observed that TH treatment induces the mRNA expression of a second enzyme of the same family, GPT (Figure S1E), which also catalyzes the reversible transamination between alanine and α-KG to form pyruvate and glutamate (Yang et al., 2002). Taken together, the above results suggest that TH activation participates in the metabolic regulation of glutamine metabolism via the induction of GPT2.

### TH induces transcription of glutamate pyruvate transaminase 2

**Figure 2:**
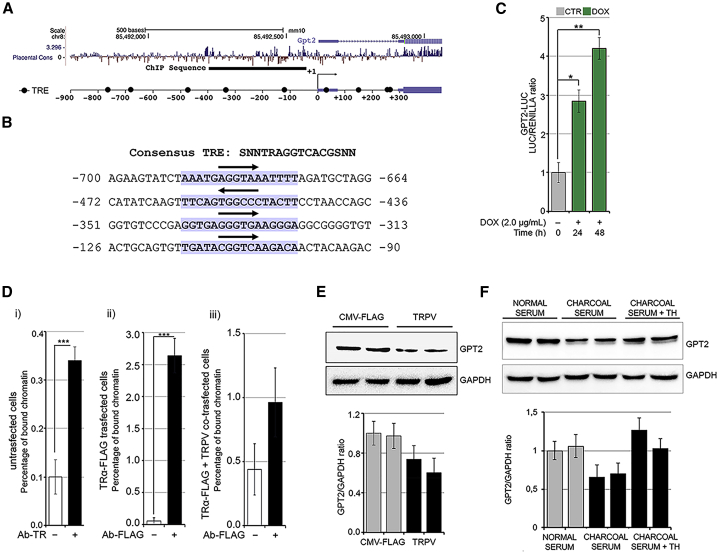
Thyroid hormone positively regulates *GPT2* transcription in muscle cells (A) The mammalian conservation, the gene structure, and the position from Genome Browser are indicated for the *GPT2* gene. The black bar represents the sequence analyzed by chromatin immunoprecipitation (ChIP). Black circles represent potential TH responsive elements (TREs) identified by *in silico* analysis. The transcription start site (TSS) is set at +1. (B) Sequence of first four consensus TREs identified upstream of the TSS. Sequence matching the consensus is highlighted in purple, and the region corresponding to the conserved AGGTCA is indicated by an arrow. (C) T3 transactivation activity was measured using the reporter plasmid GPT2-luciferase (LUC). C2C12 pTRE-D2 cultured with 2 μg/mL DOX for 24 and 48 h were transfected with the GPT2-LUC and CMV-Renilla as an internal CTR. The results are shown as means ± standard deviation (SD) of the LUC/Renilla ratios from at least 3 separate experiments, performed in duplicate. (D) ChIP assay was performed in (1) C2C12 cells using the anti-TH receptor antibodies, (2) C2C12 cells transfected with TRα-FLAG using the anti-FLAG antibody, and (3) C2C12 cells co-transfected with TRα-FLAG + the dominant-negative of TH receptor (TRPV) using the anti-FLAG antibody. (E) Protein levels of GPT2 were measured by western blot analysis in C2C12 cells transfected with TRPV or CMV-FLAG for 48 h. (F) Protein levels of GPT2 were measured by western blot analysis in C2C12 cells cultured in normal serum, charcoal serum, and charcoal serum + TH for 48 h. The histograms below show the quantification of the GPT2 protein levels versus GAPDH levels. Data represent the mean ± SD of the mean of fifteen replicates. ^∗^p < 0.05, ^∗∗^p < 0.01, ^∗∗∗^p < 0.001.

To determine whether GPT2 is directly regulated by THs via a TH responsive element (TRE), we analyzed *in silico* a DNA region upstream of *GPT2* 5′ UTR comprising sequences from −900 to +300 bp relative to the transcriptional start site (TSS) of the *GPT2* gene (Figure 2A). This analysis revealed nine potential consensus TRE binding sites, among which the first four sites upstream of the TSS are shown, as an example, in Figure 2B. We cloned the promoter region of mouse *GPT2* that contained a 1.2 kb fragment (from −900 to +300 bp relative to TSS) into the pGL3b luciferase reporter construct. Transient transfection in C2C12 cells demonstrated that THs induce *GPT2* promoter activation in DOX-treated pTRE-D2 cells (Figure 2C). Furthermore, a chromatin immunoprecipitation assay (ChIP) confirmed that the thyroid receptor (TR) physically binds to the *GPT2* promoter region (Figure 2D). The physical binding was confirmed in C2C12 cells transiently transfected with a FLAG-TRα plasmid, in which we observed that the FLAG antibody immunoprecipitates the *GPT2* promoter region, but this effect is lost in cells co-transfected with the TR dominant-negative isoform (TRPV [Zavacki et al., 1993]; Figure 2D). To verify that the positive regulation of GPT2 expression requires TR binding to the *GPT2* promoter in muscle cells, we transfected C2C12 cells with the TRPV plasmid or the empty vector (CMV-FLAG). We also cultured C2C12 cells in a TH-depleted medium with charcoal-stripped serum or normal serum. As expected, basal levels of GPT2 were reduced by TRPV transfection (Figure 2E), and, notably, TH deprivation in charcoal medium also reduced basal GPT2 levels, while TH replacement induced them (Figure 2F). Taken together, these data demonstrate that TH mediates GPT2 expression via a proximal, upstream TRE.

### TH induces glutamine deamination flux and increases glutamine addiction of muscle cells

**Figure 3:**
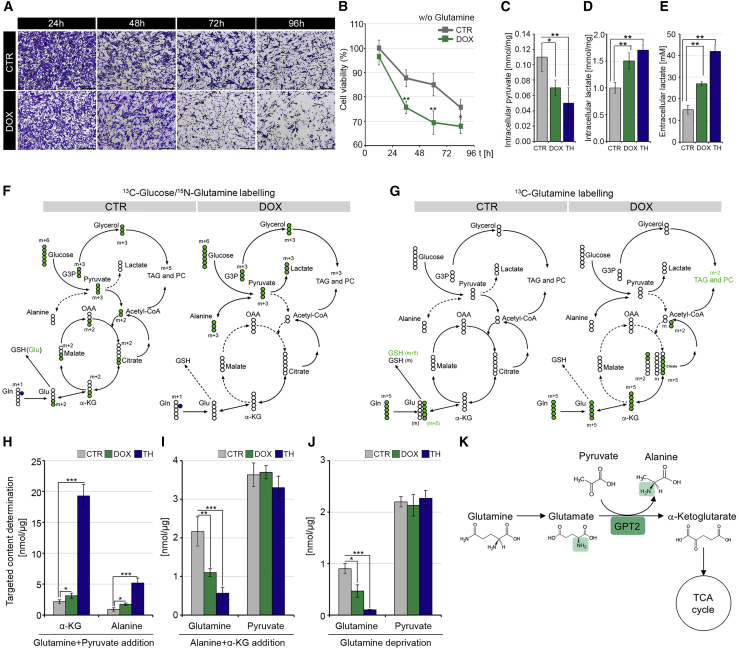
Thyroid hormone activation induces glutamine addiction (A) Representative images of C2C12 pTRE-D2 cells cultured in the presence (DOX) or absence (CTR) of 2 μg/mL DOX in DMEM without glutamine at different time points (24, 48, 72, and 96 h). Scale bar, 50 μm. (B) Cell viability percentage of the same cells as in (A) was determined by MTT assay. (C–E) Intracellular pyruvate (C), intracellular lactate (D), and extracellular lactate (E) concentrations were measured in C2C12 pTRE-D2 cells cultured for 48 h in the presence of DOX, THs, and in untreated CTR cells by GC-MS analysis. The results are shown as means ± SD from at least 3 separate experiments. (F and G) Schematic of isotopologue distributions of metabolites derived from either ^13^C-labeled glucose, ^15^N-labeled glutamine, (F) or ^13^C-labeled glutamine (G) in C2C12 pTRE-D2 cells cultured for 48 h in the presence (+DOX) or absence (–DOX) of doxycycline by stable-isotope-resolved metabolomic analysis. Empty circles represent unlabeled atoms; green circles represent labeled carbon (^13^C), and blue circles represent labeled nitrogen (^15^N). The arrows indicate the flux of reactions in each of the metabolic pathways. The mass increase (m+1, m+2, m+3, m+5, and m+6) indicates the transfer of labeled atoms among metabolites. G3P, glyceraldehyde-3-phosphate; TAG, triacylglycerol; PC, phosphatidylcholine; α-KG, alpha-ketoglutarate; Glu, glutamate; Gln, glutamine; GSH, glutathione; OAA, oxaloacetate. (H–J) Intracellular levels of α-KG, alanine, pyruvate, and glutamine extracted from DOX- or TH-treated C2C12 pTRE-D2 compared with untreated CTR cells and cultivated in a medium containing an excess of either pyruvate and glutamine (H), α-KG and alanine (I), or in the absence of glutamine. Data represent the mean ± SD of the mean of fifteen replicates. ^∗^p < 0.05, ^∗∗^p < 0.01, ^∗∗∗^p < 0.001. (K) Schematic of GPT2-mediated coupling of glutamine-driven TCA anaplerosis.

We asked if, by up-regulating GPT2, THs induce metabolic reprogramming thereby increasing the glutamine dependence of muscle cells. C2C12 TET-ON D2 cells were cultured in a glutamine-depleted medium for 96 h, and cell viability was measured by crystal violet and MTT assays (Figures 3A and 3B). Notably, glutamine deprivation reduced cell viability in the control C2C12 cells, and this effect was strongly enhanced by D2 induction, which suggests a glutamine addiction of D2-overexpressing cells.

GPT2 catalyzes the conversion of glutamate + pyruvate into α-KG and alanine in both a direct and reverse direction, thus driving the dynamic coupling of pyruvate production to glutamine catabolism or vice versa. Thus, we investigated the fate of labeled glucose in the cells upon THs treatment or D2 induction. To this aim, we measured the concentration of pyruvate and lactate, which are the main products of the glycolytic pathway. Interestingly, intracellular pyruvate was reduced, and intracellular and extracellular lactate were increased, in D2-overexpressing and TH-treated cells (Figures 3C–3E).

We reconstructed the flux of reactions involving glucose and glutamine utilization that were altered by D2 overexpression using a stable-isotope-tracing analysis with either ^13^C-labeled glucose, ^13^C-labeled glutamine, or ^15^N-labeled glutamine. Overexpression of D2 and treatment with TH resulted in a rapid increase of all labeled metabolite uptakes (Figures S2A–S2C). Chasing ^13^C-labeled glucose, we observed that D2 induction increased glycolysis and ^13^C-lactate production as shown by the levels of the m+3 isotope of lactate (lactate m+3) (Figures 3F and S2D). Interestingly, we observed that the production of lactate accounts for only part of the glucose consumed, as witnessed by an increase in the levels of the labeled alanine (alanine m+3; Figures 3F and S2E). The newly synthesized alanine derives from pyruvate conversion, which is further supported by an α-KG (m+2), malate (m+2), and citrate (m+2) reduction (Figures S2F–S2H). These data suggest that D2 promotes a transamination reaction that produces alanine and α-KG from pyruvate and glutamate (Figures 3F and S2D–S2H). This was indeed confirmed by tracing ^13^C-glutamine (Figure 3G) and ^15^N-glutamine. Upon D2 overexpression or TH treatment, glutamine is first converted into glutamate, to be next transformed into α-KG by the GPT2 enzyme, as shown by the increase in glutamate (m+5) and α-KG (m+5) (Figures S2J and S2K). Rather than continuing along the Krebs cycle, α-KG (m+5) is ultimately converted into citrate, as shown by the increased citrate (m+5) and by the concomitant reduction in citrate (m+2) and malate (m+4) (Figures S2L and S2M). Finally, both ^13^C-glucose and ^13^C-glutamine tracing experiments clearly agreed in showing that D2 overexpression (as well as TH treatment) conveys glutamine carbon into lipogenesis for sustaining plasma membrane lipids and triacylglycerol (TAG) synthesis, as confirmed by the increase in labeled phosphatidylcholines (PCs) (Figures S2I and S2N). These data further support the message of paper, i.e., that D2 promotes reductive carboxylation to produce citrate from glutamine (Figures 3F and 3G).

The transamination catalyzed by GPT2 is a reversible reaction. Notwithstanding, our metabolomic profiling indicates that D2 overexpression preferentially induces the production of α-KG. To verify if this preferential direction of a TH-induced, GPT2-catalyzed reaction was dependent on intracellular availability of its substrates, we metabolically profiled cells grown in an excess of either α-KG and alanine or glutamine and pyruvate. Notably, independently from the availability of substrates, D2 overexpression only forces GPT2 to produce α-KG (Figures 3H and 3I). These results were also confirmed in condition of glutamine starvation, when, to simulate the complete absence of one of the GPT2 substrates, glutamine was removed from the culture medium. Even in the absence of glutamine, D2 overexpression does not induce glutamine production from α-KG (Figure 3J). Altogether, these experiments indicate that D2 overexpression (or TH treatment) directs a GPT2-dependent monodirectional conversion of glutamine into α-KG.

The above results demonstrate that GPT2 induction via THs culminates in alanine and α-KG production while consuming glutamine and pyruvate (Figure 3K), in line with the MS analysis shown in Figure 1B.

### TH up-regulates GPT2 in muscle *in vivo*

**Figure 4:**
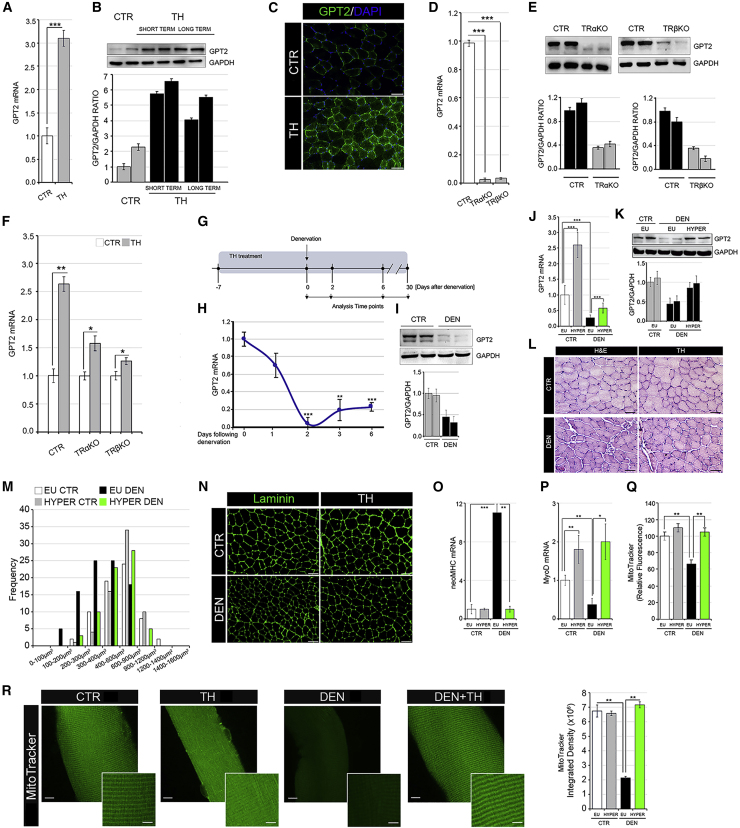
Thyroid hormone promotes GPT2 expression in *in vivo* muscles, ameliorating muscle response to denervation (A and B) Real-time PCR (A) and western blot (B) analyses of GPT2 in GC muscles of euthyroid (CTR) and hyperthyroid mice (short and long term). (C) Immunofluorescence of GPT2 protein in CTR and hyperthyroid (TH) tibial anterior (TA) muscles. Scale bar, 50 μm. (D) Basal mRNA expression levels of the *GPT2* gene were measured in GC muscles collected by WT (CTR), TrαKO, and TRβKO mice. (E) Protein levels of GPT2 were measured in the same muscles as in (D). Relative quantification of the GPT2 protein levels versus GAPDH levels is represented by histograms below. (F) GPT2 mRNA levels were measured by real-time PCR in euthyroid and hyperthyroid CTR, TRαKO, and TRβKO GC muscles. (G) Time-line of TH treatment and denervation experiments. (H) mRNA expression levels of GPT2 gene were measured in innervated (CTR) and denervated (DEN) GC muscles after 1, 2, 3, and 6 days of sciatic nerve cut. (I) Western blot analysis of GPT2 in GC CTR muscles and in GC muscles 6 days following denervation (DEN). GAPDH was used as a loading CTR. (J) mRNA expression levels of GPT2 gene were measured in non-denervated (CTR) and denervated (DEN) GC muscles from euthyroid or hyperthyroid mice (EU and HYPER) 6 days following denervation. (K) Western blot analysis of GPT2 in non-denervated (CTR) and denervated (DEN) GC muscles from euthyroid or hyperthyroid mice (EU and HYPER) after 6 days of sciatic nerve cut. Histograms below show the quantification of the GPT2 protein levels versus GAPDH levels. (L and M) H&E staining (L) and cross-sectional area (CSA) (M) of TA muscles as in (J). Scale bar, 50 μm. (N) Immunofluorescence of laminin in the same TA muscles as in (J). Scale bar, 50 μm. (O and P) mRNA expression levels of neoMHC (O) and MyoD (P) were measured in the same GC muscles as in (J). Levels of indicated genes are relative to Cyclophilin-A mRNA used as an internal CTR and normalized to the CTR euthyroid muscle, set as 1. (Q) Mitochondrial activity was measured by fluorescence-activated cell sorting (FACS) using MitoTracker Red CMXRos dye in non-denervated (CTR) and denervated (DEN) GC muscles from euthyroid or hyperthyroid mice (EU and HYPER) 2 days following denervation. The bar graph shows the relative median fluorescence versus CTR muscles arbitrarily set at 100. (R) Confocal images of MitoTracker-stained extensor digitorum longus (EDL) muscle fibers derived from same mice as in (Q). Data are presented as overviews (top rows) and higher magnifications (bottom rows). Scale bars: 10 μm (top rows) and 5 μm (bottom rows). The histogram (right) shows the quantification of MitoTracker staining of muscle fibers. Data represent the mean ± SD of the mean of nine replicates. ^∗^p < 0.05, ^∗∗^p < 0.01, ^∗∗∗^p < 0.001.

Similar to the *in vitro* observations in the C2C12 cellular system (Figures 1E and 1F), the *in vivo* GPT2 levels were higher in hyperthyroid gastrocnemius (GC) muscles than in control muscles at both mRNA and protein levels (Figures 4A–4C). Consistently, GPT2 expression was abrogated in muscles isolated from TRαΚΟ and TRβΚΟ mice (Figures 4D and 4E), and, notably, its induction by THs was reduced in TRαΚΟ and TRβΚΟ mice (Figure 4F), which confirms that both TRα and TRβ contribute to TH-dependent GPT2 expression. This set of experiments reinforces our finding that the mitochondrial enzyme GPT2 is a TH target gene in muscle, which, in turn, suggests that THs play a crucial role in the regulation of glutamine metabolism.

### TH induces GPT2 in atrophic muscles and counteracts muscle fiber atrophy following denervation

Since THs induce both muscle degradation and synthesis (Goldberg et al., 1980), we asked if the induction of GPT2 by THs is part of the anabolic or catabolic action of THs in muscle. To this aim, we used muscle denervation as an experimental model of muscle atrophy in euthyroid and hyperthyroid mice (Figure 4G). Muscle atrophy was monitored by measuring the expression of two muscle-specific E3 ubiquitin ligases, Atrogin-1 and MuRF-1, whose expression is up-regulated in catabolic conditions (Bodine et al., 2001). As expected, both genes were significantly induced by denervation (Figures S3A and S3B). After denervation, GPT2 mRNA and protein were reduced in atrophic muscles (Figures 4H and 4I). This is in agreement with the concept that patients with mutated GPT2 undergo muscle atrophy (Celis et al., 2015) and suggests that GPT2 exerts a pro-hypertrophic effect in muscle. The reduction of the mitochondrial gene *GPT2* is also in agreement with the reduced levels of succinate dehydrogenase (SDH) staining in denervated muscles (Figure S3C).

Interestingly, we observed that TH treatment attenuated the downregulation of GPT2 after denervation by sustaining expression levels of GPT2 (Figures 4J and 4K). Moreover, a reduction in fiber diameter was partially attenuated by TH treatment (Figures 4L and 4M). Laminin staining confirmed the preservation of muscle mass in denervated hyperthyroid muscles (Figure 4N). Moreover, the levels of the marker of early regeneration phase (neo major histocompatibility complex [MHC]) were not up-regulated by denervation in hyperthyroid muscles as in the euthyroid muscles, while the early myogenic differentiation marker (MyoD) was sustained in TH-treated mice compared with in euthyroid muscles, which suggests an accelerated differentiation process and a faster recovery from denervation of TH-treated muscle fibers 6 days after denervation (Figures 4O and 4P). Also, mitochondrial function, which was drastically reduced upon denervation in control mice, was rescued by TH treatment in hyperthyroid muscles and fibers (Figures 4Q and S3D). Mitochondrial content, assessed by Mito-Tracker staining, was potently enhanced by THs in both control and denervated muscle fibers (Figure 4R). Accordingly, the expression of PGC-1α, which is a mitochondrial biogenesis marker and a TH target gene (Wulf et al., 2008), was higher in denervated hyperthyroid muscles than in euthyroid denervated muscles (Figure S3E). The expression of the uncoupling gene UCP-3 paralleled that of PGC-1α (Figure S3E), while the mitochondrial fusion and fission genes and the mitochondrial respiration genes were reduced by denervation and did not change with TH treatment (Figures S3F and S3G).

**Figure 5:**
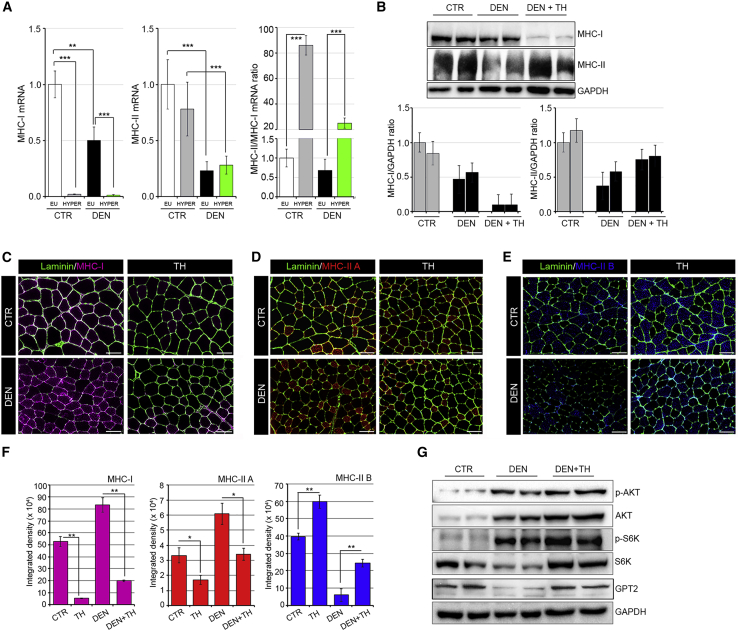
MHC profiling in euthyroid and hyperthyroid muscles during denervation-induced atrophy (A) mRNA expression levels of MHC class I and MHC class II were measured in euthyroid and hyperthyroid innervated GC muscles (CTR EU and HYPER) and after 6 days of denervation (DEN EU and HYPER). MHC class II/MHC class I mRNA ratio indicates a shift from type I to II fibers in hyperthyroid muscles (CTR HYPER and DEN HYPER). Levels of indicated genes are relative to Cyclophilin-A mRNA used as an internal CTR and normalized to the CTR euthyroid muscle. (B) Western blot analysis of MHC-slow (top) and MHC-fast (bottom) in euthyroid CTR, euthyroid denervated (DEN), and hyperthyroid denervated (DEN + TH) GC muscles after 6 days of sciatic nerve cut. Histograms below show the quantification of the indicated protein levels versus GAPDH levels. (C–E) Immunofluorescence of MHC class I (C), MHC class IIA (D), and MHC class IIB (E) in euthyroid and hyperthyroid innervated TA muscles (CTR and TH) and after 6 days of denervation (DEN and DEN + TH). Scale bar, 50 μm. (F) Relative quantification of the mean integrated density of the fluorescence signal in (C)–(E). (G) Western blot analysis using the same samples reported in Figure 4K of total and phosphorylated Akt, total and phosphorylated S6K, and GPT2 proteins. Data represent the mean ± SD of the mean of nine replicates. ^∗^p < 0.05, ^∗∗^p < 0.01, ^∗∗∗^p < 0.001.

Notably, TH treatment caused a major shift in the fiber type proportion from slow type I fibers to fast type II fibers in both denervated and control muscles as demonstrated by the mRNA levels of the MHC isoform profile (Figure 5A). In detail, THs potently reduced the levels of the MHC class I mRNA, thus affecting the relative proportion of MHC class II/MHC class I. The same shift was confirmed by western blot and immunofluorescent analysis of MHC protein levels (Figures 5B–5F).

We next measured the expression of key regulators of the anabolic pathways in muscle such as the Akt/mTOR pathway, whose expression has been shown to promote protein synthesis in muscle as adaptive responses to denervation (Castets et al., 2019). Levels of pAkt (Ser473) were higher in the GC muscle of TH-treated mice than in denervated euthyroid muscles (Figures 5G, S4A, and S4B). Accordingly, the levels of p-S6 kinase (p70S6K1) were also higher in the GC muscle of denervated hyperthyroid mice than in denervated euthyroid muscles (Figures 5G, S4A, and S4B). Levels of p-Akt and p-S6K were also up-regulated by THs in control, non-denervated muscles, thus showing that THs promote the p-Akt pathway in basal conditions (Figure S4B). These results demonstrate that both mTORC1 and Akt, which are activated in muscle after nerve injury (Castets et al., 2019), are also positively regulated by THs, which confirms that THs potentiate muscle recovery following denervation.

Lastly, to identify the endpoint of recovery from muscle denervation in TH-treated muscles, we analyzed muscles at various time points post-denervation. As shown in Figure S5, laminin expression and fiber diameters were higher in hyperthyroid muscles than in euthyroid muscles 30 days after denervation. However, a subgroup of small-diameter fibers, with centrally located nuclei and that are MHC-class-I-positive, appear in the TH-denervated muscles, leading to the speculation that long-term exposure to THs increases the diameter of fibers that are MHC-class-I-negative (Figure S5C).

Taken together, these results suggest that THs, via GPT2 induction, can improve muscle regeneration processes by promoting a rapid complete recovery of muscle fibers at late stages of denervation.

### Amino acid supplementation protects from muscle atrophy without inducing a muscle fiber shift

**Figure 6:**
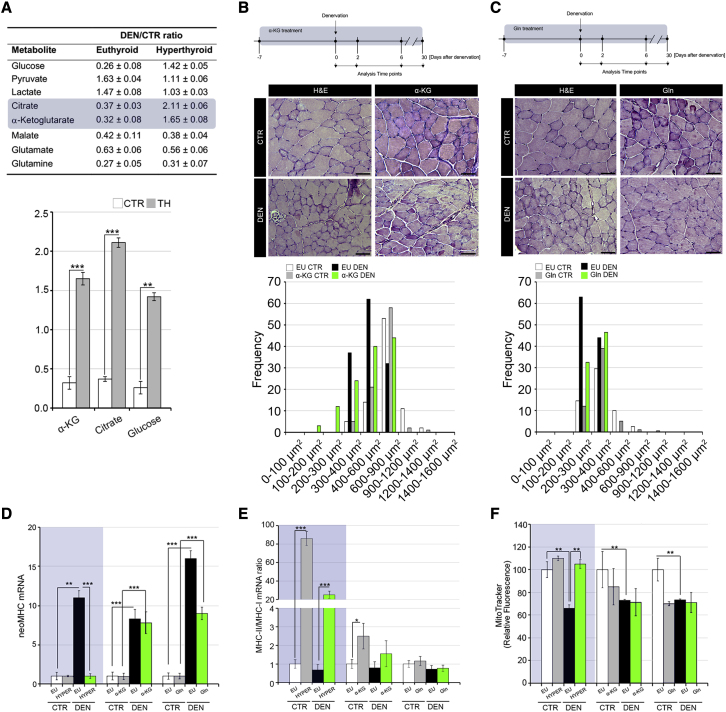
Amino acid supplementation protects from denervation-induced atrophy without promoting muscle fiber shift (A) The most significantly altered metabolites resulting from metabolomic analysis of GC muscles are shown in the table. The denervated/innervated ratio is represented in euthyroid and hyperthyroid GC muscles. The denervated/innervated ratio of α-KG, citrate, and glucose levels in euthyroid and hyperthyroid conditions are shown below. (B and C) Timeline of α-KG (left) and Gln (right) supplementation in denervation experiments. H&E staining of innervated CTR and denervated TA muscles from α-KG- (B) and Gln (C)-supplemented mice. Scale bar, 50 μm. The graphical representation of CSA of TA muscle fibers is shown below each H&E panel. (D and E) mRNA expression levels of neoMHC and the ratio MHC class II/MHC class I were measured in innervated CTR and denervated GC muscles. Each graph shows TH-treated (left, gray box), α-KG-supplemented (middle), and Gln-supplemented (right) GC muscles. Levels of indicated genes are relative to Cyclophilin-A mRNA used as an internal CTR and normalized to the CTR muscle. (F) Mitochondrial activity was measured by FACS using MitoTracker Red CMXRos dye in innervated CTR and denervated GC muscles as in (D) and (E). The bar graph shows the relative median fluorescence versus CTR muscles arbitrarily set at 100. Data represent the mean ± SD of the mean of nine replicates. ^∗^p < 0.05, ^∗∗^p < 0.01, ^∗∗∗^p < 0.001.

To gain further insight into the TH-regulated metabolic alterations induced in muscle atrophy, we analyzed the metabolic profile of denervated and control muscles in euthyroid and hyperthyroid conditions. GC-MS analysis revealed that, similar to what was also observed in muscle cells (Figure 1B) during muscle denervation, α-KG and citrate levels were higher in TH-treated muscles than in euthyroid muscles (Figure 6A). Given that amino acid supplementation is widely used to treat muscle wasting syndromes (Cruzat and Tirapegui, 2009), we compared the anti-atrophic action of THs with that promoted by amino acid supplementation and performed mice denervation in the presence of α-KG or glutamine (Gln) (Figures 6B and 6C). As expected, both α-KG and Gln supplementation reduced the fiber diameter loss post-denervation (Figures 6B and 6C). Notably, the key markers of fiber-type shifts altered by THs (reduced neoMHC and a shift from type I to II fibers) were unchanged by α-KG or Gln supplementation, which suggests that the positive effect of α-KG and Gln on the response to denervation did not involve structural muscle composition and GPT2 expression (Figures 6D, 6E, S6A, and S6B). Interestingly, the mitochondrial function, which was drastically reduced upon denervation in control mice and rescued by TH treatment, was not rescued in either α-KG- or Gln-treated muscles (Figures 6F and S6C). These results suggest that TH signaling improves post-denervation muscle recovery by affecting multiple aspects of muscle fiber composition. In fact, TH signaling acts as a metabolic determinant via GPT2 induction, regulates myofiber differentiation by inducing myogenic markers, and determines the structural components by promoting a shift toward differentiated muscle fibers.

### GPT2 is the main mediator of the TH-dependent pro-anabolic action after denervation

**Figure 7:**
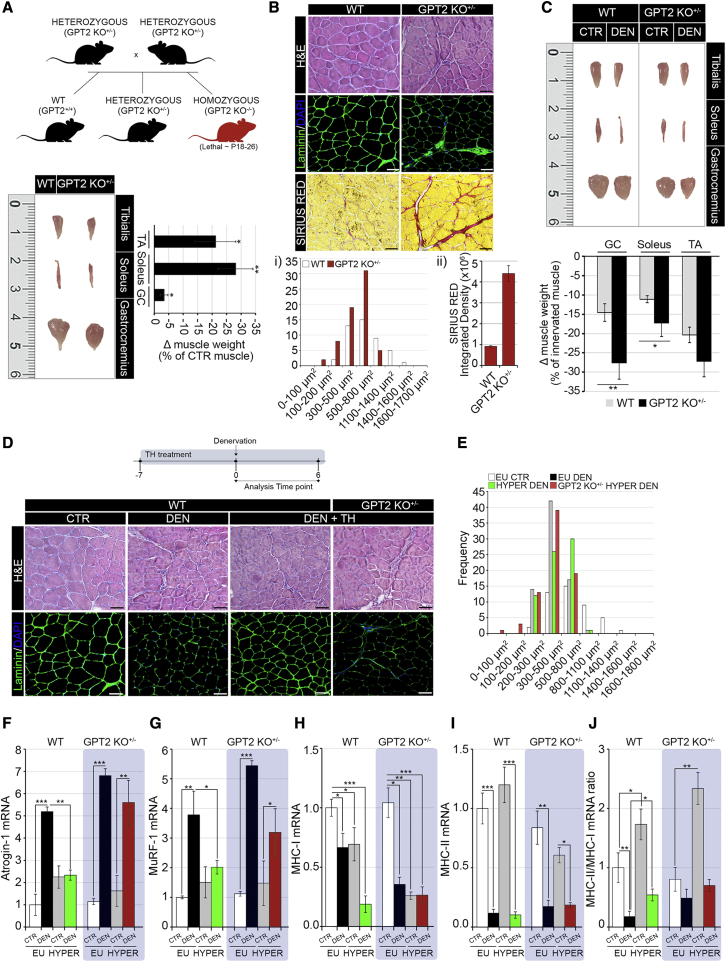
GPT2 is the critical mediator of pro-anabolic action of thyroid hormone in denervation-induced atrophy (A) Diagrammatic representation of breeding for the generation of GPT2 KO mice and pictures of TA, soleus, and GC of GPT2 KO^+/–^ and WT mice together with a graphical representation of the loss (Δ) muscle weight expressed as the percentage compared with WT. (B) H&E, laminin immunofluorescence, and Sirius red staining of GPT2 KO^+/–^ and WT TA muscles. Scale bar, 50 μm.The CSA (1) and quantification of the Sirius red density (2) relative to WT muscles is reported below. (C) Pictures of TA, soleus, and GC muscles after 6 days of denervation from GPT2 KO^+/–^ and WT mice. (Bottom) The graphical representation of Δ muscle weight expressed as the percentage compared with the innervated contralateral muscle in the same mouse. n ≥ 8 mice for GPT2 KO^+/–^ and WT CTR littermates. (D) Timeline of TH treatment and denervation experiments in GPT2 KO^+/–^ and WT mice. H&E and laminin staining of euthyroid denervated (DEN) and hyperthyroid denervated (DEN + TH) WT and GPT2 KO^+/–^ mice with respect to CTR euthyroid innervated TA muscles. Scale bar, 50 μm. (E) CSA of the same muscles as in (D) (F–J) mRNA expression levels of the indicated genes and MHC class II/MHC class I mRNA ratio in GC muscles as in (D). Levels of indicated genes are relative to Cyclophilin-A mRNA used as an internal CTR and normalized to the CTR euthyroid muscle. Data represent the mean ± SD of the mean of nine replicates. ^∗^p < 0.05, ^∗∗^p < 0.01, ^∗∗∗^p < 0.001.

In order to validate GPT2 as a TH-dependent, pro-hypertrophic gene in skeletal muscle, we sought to analyze the effects of TH treatment in a mouse with a genetic loss of function of GPT2 (Ouyang et al., 2016). GPT2-null mice have been shown to be affected by neurological and neuromotor dysfunctions (Ouyang et al., 2016); however, the muscle phenotype of GPT2-null mice has never been described. We investigated the muscle phenotype of mice with a heterozygous loss of the *GPT2* gene (GPT2 KO^+/–^), since the homozygous deletion of GPT2 is lethal at post-natal days 18–26 (P18–P26) (Ouyang et al., 2016) (Figure 7A). In these mice, GPT2 expression was drastically reduced at the mRNA and protein levels in muscle fibers (Figures S7A and S7B), and TH treatment increased GPT2 expression over the basal levels (Figures S7A and S7B). Interestingly, GPT2 KO^+/–^ muscles were characterized by slightly reduced muscle mass (Figure 7A) and a more pronounced alteration of muscle fiber diameter, associated with reduced laminin content (1) and muscle fibrosis (2) (Figure 7B). The atrophic condition was even more exacerbated by muscle denervation that resulted in a drastic loss of muscle weight (Figure 7C). To test if GPT2 is the main mediator of the TH-dependent, pro-hypertrophic function following denervation, we treated wild-type (WT) and GPT2 KO^+/–^ mice with THs for 1 week before denervation (Figure 7D). Notably, while THs protected muscles from a massive loss of muscle mass, this effect was drastically reduced in GPT2 KO^+/–^ mice (Figures 7D and 7E). Consistently, the expression of the atrogenes MuRF-1 and Atrogin-1 was attenuated in hyperthyroid denervated muscles, while the loss of GPT2 rescued the TH-dependent muscle wasting protection (Figures 7F and 7G). Moreover, while the loss of GPT2 attenuated the TH-dependent, anti-atrophic program, it did not affect the proportion of myosin heavy chain isoforms (Figures 7H–7J). Together, these results confirm that GPT2 is a crucial mediator of the TH-dependent anabolic function.

## Discussion

Herein, we describe specific functions of TH signaling in muscle Gln metabolism that affect muscle homeostasis and muscle fiber atrophy. Mostly synthetized by skeletal muscle, Gln is used by the kidney for acid-base homeostasis and by the liver for urogenesis and gluconeogenesis (Neu et al., 1996). Skeletal muscle is an example of high Gln synthetizing tissue that becomes critically important in catabolic conditions (Owen et al., 2002). Gln exerts pleiotropic functions in cells. In rapidly proliferating cells, it plays an anaplerotic role by replenishing tricarboxylic acid (TCA) cycle intermediates (Daye and Wellen, 2012); it is also a cellular carbon source for fatty acid synthesis as well as a source of nitrogen for the synthesis of purines, pyrimidines, aminosugars, neurotransmitters, and GSH (Tapiero et al., 2002). Consequently, Gln is required for many metabolic pathways generally known as “glutaminolithic pathways” (Newsholme et al., 2003).

By matching metabolomic approaches with gene regulation studies, we found that the intracellular activation of THs augments the reductive Gln metabolism mediating a switch in carbon source that prevents muscle atrophy during catabolic conditions.

Metabolomics analysis in muscle cells demonstrated that THs modulate the Gln metabolism and that the *GPT2* gene mediates this effect. In detail, THs induce the transcription of GPT2, which catalyzes the reversible transamination between pyruvate and glutamate to form alanine and α-KG (Ouyang et al., 2016). In skeletal muscle, THs are key regulators of myocyte differentiation (Dentice et al., 2010; Salvatore et al., 2014), post-damage regeneration of myofibers (Dentice et al., 2014; An et al., 2021), and the redox state (Sagliocchi et al., 2019). THs also regulate the myosin heavy chain switch and the rate of contraction and relaxation of muscle fibers (Simonides and van Hardeveld, 2008). Glut4 is a positive target of THs, which is consonant with the pro-insulinemic effects of THs in skeletal muscle (Wang et al., 2018).

Our finding that *GPT2* is a TH target gene in muscle adds additional insights into the critical role of THs as physiological endocrine regulators of muscle homeostasis. We show that THs regulate Gln and glucose metabolism through GPT2 and couple glycolysis to TCA cycle anaplerosis. Recessive mutations in the *GPT2* gene have been described in a human neurological syndrome involving intellectual and developmental disability (IDD), post-natal microcephaly, and spastic paraplegia, coupled with small stature (Celis et al., 2015; Hengel et al., 2018; Ouyang et al., 2016). Similarly, mice with GPT2 loss are characterized by intellectual disability, reduced brain growth, and altered motor symptoms (Ouyang et al., 2016).

Our study describes a muscle phenotype that can be added to the GPT2-related disease. Analysis of mice with a heterozygous germline mutation in the *GPT2* gene (Ouyang et al., 2016) revealed a drastic reduction of fiber diameter, enhanced fibrotic infiltration of muscle fibers, and a reduced muscle weight. These muscular aspects could be caused by both of the neuromotor alterations following the altered neuronal circuitry, synaptogenesis and myelination, but could be also attributed to the intramuscular metabolic adaptations following GPT2 loss of function that can contribute to the unbalanced anabolic/catabolic ratio in these mice. Indeed, when challenged with muscle denervation, GPT2^+/−^ mice underwent profound muscle loss when compared with WT mice.

### TH-induced GPT2 up-regulation contrasts muscle loss during denervation

To assess the role of TH-induced GPT2 in muscle atrophy, we investigated if the GPT2 induced by THs is involved in metabolic reprogramming during muscle catabolic conditions. We used muscle denervation as a model of muscle atrophy. First, we observed that GPT2 expression together with mitochondrial mass and activity are reduced during atrophy and that TH treatment prevents GPT2 down-regulation and attenuates mitochondrial down-modulation. These effects resulted in the prevention of fiber-diameter reduction and in a partial resistance to the atrophic process.

During muscle atrophy, the endogenous amino acid pool is maintained predominantly by proteolysis, and muscle catabolism exceeds anabolic processes, thereby the decline in GPT2 expression that we observed in denervated muscles is in line with the degenerative phase of muscle fibers following denervation. From this point of view, the ability of THs to sustain GPT2 expression in denervated muscles confirms that this process is part of the pro-anabolic function of THs. Notably, the muscle loss prevention promoted by THs was not limited to metabolic regulation but also involved the muscle fiber shift from low oxidative type I fibers to twitch glycolytic type II fibers, a process that was paralleled by increased levels of the myogenic proteins MyoD and Myogenin.

Given the above, we conclude that THs attenuate muscle degradation both by regulating the Gln pathways and by increasing the differentiation and maturation of muscle fibers after denervation. This effect is more potent than supplementation with amino acids such as Gln or α-KG, which are often used to treat muscle atrophy (Jang et al., 2015; Cruzat and Tirapegui, 2009). While THs protect the atrophic muscle from massive muscle loss consequent to denervation, similar to what occurs after amino acid supplementation, a major difference between the two effects is that THs, but not amino acid supplementation, are potent inducers of myosin shift, and potentiate the formation of fast-twitch fibers, and are thus muscle-sustaining factors. Thus, THs can profoundly impact muscle forces and functionality in pathophysiological conditions such as those following acute exercise or muscle wasting syndromes.

### GPT2 is essential for the pro-anabolic function of TH

To investigate the contribution of GPT2 in the TH-dependent attenuation of acute muscle loss following denervation, we induced a systemic hyperthyroidism in GPT2 KO^+/–^ mice in which the basal expression of GPT2 was elevated in muscle fibers due to the TH treatment (Figures S7A and S7B). Those experiments led to different observations (Table S1): (1) analyzing the cross-sectional area (CSA) profile and fiber phenotype of non-denervated, control muscles, we observed that the TH treatment did not cause alterations of fiber diameters, thus showing that our experimental conditions are in a physiological window in which muscle atrophy and hypertrophy are in equilibrium and THs induce both muscle loss and synthesis. However, upon denervation, in which GPT2 expression drastically drops, the TH treatment, causing GPT2 re-expression, acts as pro-hypertrophic agent (Figures 4J–4N). (2) Strikingly, while hyperthyroid denervated muscles were partially protected by acute muscle loss, the heterozygous depletion of GPT2 (in GPT2 KO^+/–^ hyperthyroid denervated muscles) did not prevent muscle loss, demonstrating that GPT2 is the main mediator of the TH-dependent pro-anabolic action (Figures 7D and 7E). (3) The GPT2 KO^+/–^ hyperthyroid denervated muscles, however, did not show severe acute muscle loss as the GPT2 KO^+/–^ euthyroid denervated muscles, thus suggesting that THs improve the atrophic phenotype of GPT2 KO^+/–^ muscles over their basal level (Figure S7); we argue that THs potentiate the expression of GPT2 in the WT allele of GPT2 KO^+/–^ mice. This speculation suggests that THs can be viewed as therapeutic tools against muscle loss in conditions of altered Gln metabolism as in the case of GPT2 KO^+/–^ mice.

In conclusion, our data show that THs are fine regulators of the balance between muscle mass loss and synthesis. Indeed, while short-term TH treatment did not affect fiber diameter in normal non-atrophic muscles, surprisingly, it attenuated muscle wasting and preserved fiber integrity in atrophic muscles. This effect is in contrast with the general described pro-atrophic action of THs and with the observation that an excess of THs in hyperthyroid patients is associated with muscle weakness and increased Ca^2+^ recycling (Bloise et al., 2018; Brennan et al., 2006; Muller and Seitz, 1984; Riis et al., 2005). We interpreted the action of the TH-GPT2 axis as part of the pro-hypertrophic programs triggered by THs, which indeed, together with accelerated catabolism, are involved in the promotion of futile cycles (Vaitkus et al., 2015), which is also underlined by the fact that muscle weakness and fatigue are common to both hypo- and hyperthyroidism in humans (Argov et al., 1988). Finally, beside the skeletal muscle, the TH-GPT2-anaplerosis axis could be part of the pro-glycolitic and pro-Warburg effects exerted by TH and GPT2 in cancer, in which anaplerosis is a keystone of the metabolic reprogramming of cancer cells (Miro et al., 2021; Smith et al., 2016).

### Limitations of the study

The main message of our study is that TH coordinates Gln metabolism and anaplerotic process through the transcriptional control of GPT2 in skeletal muscle. Notably, patients with GPT2 loss of function are affected by muscle atrophy and weakness, in line with the pro-hypertrophic function of GPT2. However, there is still missing information about the physio-pathological implications of the TH-GPT2 axis in muscle. For instance, we used the sciatic nerve rescission as a model of neuromuscular atrophy. However, additional models of muscle atrophy such as cancer cachexia, fasting, and sarcopenia should be tested in order to gain insights into the general effects of GPT2 in muscle wasting syndromes. Furthermore, while we demonstrate that THs sustain GPT2-dependent anabolic action, thus suggesting that THs could represent a valid treatment in clinical conditions of protein deprivation as in the case of GPT2-null patients, we did not investigate the effects of TH treatments in GPT2-null mice due to the lethality of homozygous GPT2 mutations in mice. Thus, further study of TH treatments in human GPT2-null patients can illuminate the underlying effects of TH in those patients.

## STAR★Methods

### Key resources table

**Table:** 

REAGENT or RESOURCE	SOURCE	IDENTIFIER
**Antibodies**		

Rabbit polyclonal anti-GPT2	Proteintech	16757-1-AP; RRID:AB_2112098
Rabbit polyclonal anti-Akt	Cell Signaling	#9272; RRID:AB_329827
Rabbit polyclonal anti-Phospho-Akt (Ser473)	Cell Signaling	#9271;RRID:AB_329825
Rabbit polyclonal anti-p70 S6 Kinase	Cell Signaling	#9202; RRID:AB_331676
Rabbit polyclonal anti-Phospho-p70 S6 Kinase (Thr389)	Cell Signaling	#9205; RRID:AB_330944
Mouse monoclonal anti-Myosin (Skeletal, Slow)	Sigma-Aldrich	M8421;RRID:AB_477248
Mouse monoclonal anti-Skeletal Myosin (FAST)	Sigma-Aldrich	M4276;RRID:AB_477190
Mouse monoclonal anti-Myosin Heavy Chain Type IIA	DSHB	SC-71; RRID:AB_2147165
Mouse monoclonal anti-Myosin Heavy Chain Type IIB	DSHB	BF-F3; RRID:AB_2266724
Goat anti-Mouse IgG (H + L) Alexa Fluor 488	Invitrogen™	A-11001; RRID:AB_2534069
Goat anti-Mouse IgM (Heavy chain) Alexa Fluor 555	Invitrogen™	A21426; RRID:AB_2535847
Rabbit polyclonal anti-Laminin	Abcam	Ab11575; RRID:AB_298179
Rabbit polyclonal anti-GAPDH	Elabscience	E-AB-20059; RRID:AB_2905551
Mouse monoclonal Anti-Flag	Sigma-Aldrich	F3165; RRID:AB_259529
Anti-Thyroid hormone receptor antibody (C3)-Chip Grade	ABCAM	ab-2743; RRID:AB_2043044
Anti-Thyroid hormone receptor beta antibody-Chip Grade	ABCAM	ab-5622; RRID:AB_304991

**Chemicals, peptides, and recombinant proteins**		

Dulbecco'’s Modified Eagle Medium (DMEM)	Gibco	12491-015
Fetal Bovine Serum (FBS)	Microgem	S1810-500
Sodium pyruvate	Sigma-Aldrich	S8636
Alanine	Sigma-Aldrich	A7627-1G
Glutamine	Sigma-Aldrich	G7513
D-GLUCOSE (U-13C6, 99%)	Cambridge Isotope Laboratories, Inc.	CLM-1396
L-GLUTAMINE (AMIDE-15N, 98%+)	Cambridge Isotope Laboratories, Inc.	NLM-557
L-GLUTAMINE-13C5 99%	Cambridge Isotope Laboratories, Inc.	CLM-1822-H
Goat Serum	Sigma-Aldrich	G 9023
Tetracycline-free Fetal Bovine Serum (Tet free FBS)	Clontech	631106
Doxycycline	Sigma-Aldrich	D9891
Penicillin/Streptomycin	Gibco	15140-122
Matrigel	Corning	Cat# 354248
MCDB	Sigma	Cat# M6770
Ultroser	Pall	Cat# 15950-017
Passive Lysis Buffer	Promega	E194A
Lipofectamine 2000	Invitrogen	11668-019
Immobilon Western Chemiluminescent HPR Substrate	Millipore	WBKLS0500
Protein G Plus/Protein A Agarose Suspension	Calbiochem	IP05
Trizol Reagent	Gibco	15596018
Collagenase A	Roche	11088793001
Dispase II	Roche	#04942078001
Bis-acrylamide	Sigma-Aldrich	#M1533
TEMED	Sigma-Aldrich	#M1533
APS	Sigma-Aldrich	#A3678
Eukitt® mounting medium	Bio Optica	09-00250
3,3′,5-Triiodo-L-thyronine sodium salt (T3)	Sigma-Aldrich	T6397-1G
L-Thyroxine sodium salt pentahydrate (T4)	Sigma-Aldrich	T2501
α-Ketoglutaric acid sodium salt (AKG)	Sigma-Aldrich	K1875
AIN-93G Modified + Gln diet	Envigo	223741
MitoTracker™ Red CMXRos	Invitrogen	M7512

**Critical commercial assays**		

Immobilon Western Chemiluminescent HPR Substrate	Millipore	WBKLS0500
SYBR green master mix	Biorad	172-5121
SuperScript™ VILO™ Master Mix	Thermo Fisher	11755500
Alanine Transaminase Activity Assay kit	Abcam	ab105134
Dual-Luciferase Reporter Assay System	Promega	E1910

**Deposited data**		

Original western blot images	This paper	Mendeley Data: https://doi.org/10.17632/jfhwj4d439.1

**Experimental models: Cell lines**		

C2C12	ATCC	CRL-1772™
C2C12 TET ON D2	Laboratory of Monica Dentice	
(Sagliocchi et al., 2019)	N/A	
Primary sorted satellite cells D2WT	This paper	N/A
Primary sorted satellite cells D2KO	This paper	N/A

**Experimental models: Organisms/strains**		

C57BL/6J	Jackson Laboratory	Stock No:000664
Gpt2^tm1a(KOMP)Wtsi^ (GPT2KO^+/-^)	UC Davis, MMRRC	MGI:4363039
TRaKO and TRbKO mice	Laboratory of G. Williams, Imperial College (originally generated by J.Samarut, UMR)	N/A
Mouse Tg:Pax7-nGFP D2WT	(Sambasivan et al., 2009)	MGI:5308730
Mouse Tg:Pax7-nGFP D2KO	Laboratory of Domenico Salvatore	N/A

**Oligonucleotides**		

Individual Oligonucleotides Listed in Table S2	Eurofins	N/A

**Recombinant DNA**		

pGL3-GPT2 promoter-LUC	This paper	N/A
TR-α FLAG	Zavacki et al., 1996	N/A
TRPV	Zavacki et al., 1993	N/A

**Software and algorithms**		

Cell^∗^F Olympus Imaging Software	Olympus	www.soft-imaging.net
ImageJ 1.45s	National Institute of Health, USA	http://imagej.nih.gov/ij
X-Tile (version 3.6.1)	Camp et al., 2004	http://tissuearray.org
Leica Application Suite LAS X Imaging Software	Leica Microsystems	www.leica-microsystems.com/LAS-X
Gene Cluster 3.0	(Yeung et al., 2001)	Stanford University
Java TreeView (version 1.16r4)	(Yeung et al., 2001)	http://jtreeview.sourceforge.ne

### Resource availability

#### Lead contact

Further information and requests for resources and reagents should be directed to and will be fulfilled by the lead contact, Monica Dentice (monica.dentice@unina.it).

#### Materials availability

All unique/stable reagents generated in this study are available on request from the lead contact, without restriction.

### Experimental model and subject details

#### Cell lines and culture conditions

C2C12 cells were obtained from ATCC and cultured in Dulbecco's modified Eagle Medium (DMEM) supplemented with 10% Fetal Bovine Serum (FBS, Microgem), 2 mM glutamine, 50 i.u. penicillin, and 50 μg/mL streptomycin (proliferating medium). C2C12 TET-ON D2 cells, previously generated by Sagliocchi et al. Redox Biology 2019 (Sagliocchi et al., 2019), were cultured in DMEM supplemented with 10% tetracycline-free FBS (Clontech, Mountain View, CA, USA), 100μg/mL geneticin (Biowest, Nuaillé, France) and 0.8μg/mL puromycin (Invitrogen, Carlsbad, CA, USA). D2 expression was turned on by 2 μg/mL DOX (Clontech, Mountain View, CA, USA). For studies in proliferative conditions, C2C12 cells were grown at 40–50% confluence and treated with TH (T3 and T4, Sigma-Aldrich S. Louis, Missouri, USA) at a 30 nM final concentration for 10, 24 or 48h as indicated. For experiments in TH-depleted serum, cells were grown in Charcoal Stripped medium (CH) and, when required, treated with 30 nM TH. For glutamine deprivation, cells were cultured in glutamine- and pyruvate-free DMEM with 4.5 g/L glucose (Fisher Scientific, Catalog No. SH30081) with 10% dialyzed FBS (dFBS; Invitrogen, Catalog No. 26400). For glucose deprivation, cells were cultured in glucose-free DMEM (Invitrogen, Catalog No. A14430) with 10% of dFBS. For supplementation experiments C2C12 TET-ON D2 cells were grown in culture medium containing final concentration of α-KG [1mM] and alanine [1mM] or glutamine [5mM] and pyruvate[1mM].

FACS-sorted satellite cells (SC) were prepared from Tg:Pax7-nGFP D2 wild type or D2KO mice as previously described (Rocheteau et al., Cell 148, 112–125, 2012). In brief, muscle dissection was done with a scalpel by removing the tissue from the bone in DMEM. Muscles were then chopped and digested with collagenase 0.1% and dispase 0.1% at 37°C. SC were prepared by FACS analysis using a MoFlo (Beckman Coulter). Cells were plated on Matrigel (BD Biosciences; catalog #354234) and cultured in 1:1 DMEM:MCDB containing 20% serum FBS and 1% Ultroser G serum substitute (Pall, Catalog No. 15950-017) When required, T3 was added in culture medium at a 10 nM final concentration and cells collected for RNA extraction.

#### Mouse strains

C57BL/6J mice were obtained from Jackson Laboratory (Bar Harbor, ME, USA). Gpt2^tm1a(KOMP)Wtsi^ (GPT2 KO^+/-^) (Stock: 047980-UCD) were obtained from University of California, Davis, Mutant Mouse Resource and Research Center (Ouyang et al., 2016). The TRαΚΟ and TRβKO mice were originally generated by Jacques Samarut (UMR, ENS, Lyon) and kindly provided by Graham Williams, Imperial College, London UK, with permission from Dr Samarut. 12 weeks-old male littermates were used in this study. Animals were handled according to national and European community guidelines, and protocols were approved by the Animal Research Committee of the University of Naples “Federico II”. Mice were housed in cages containing fresh bedding under controlled conditions, namely 20–24°C, 50–60% relative humidity, and 14:10 light-dark cycle. Food and water were available *ad libitum* on all days for all animals. All animal procedures were approved by Institutional Animal Care and Use Committee (IACUC) (protocol n. 354/2019-PR).

### Method details

#### Constructs and transfections

The 1.2 kb promoter of *GPT2* was subcloned into the pGL3 vector (Promega) with XhoI and HindIII to obtain a pGL3-GPT2 promoter-LUC plasmid. Direct sequencing was carried out to verify the correct sequence (Nucleic acid sequencing, CEINGE-Biotecnologie Avanzate s.c. an r.l.). All primers used in this study are listed in Table S2. C2C12 cells were also transfected with CMV-FLAG, TRα-FLAG (Zavacki et al., 1996) or with dominant negative of TH receptor (TRPV) (Zavacki et al., 1993) alone or in combination. Transfection experiments were performed using Lipofectamine 2000 (Life Technologies, Carlsbad, CA, USA) according to the manufacturer's instructions.

#### In silico promoter analysis

Consensus TR binding sites (Matrix ID #M00239) with similarity of 0.73 or greater in the upstream region of the mouse GPT2 gene (0.9 kb of the promoter region and 0.3 kb after the TSS) were identified using TFBIND (Table S3) (http://tfbind.hgc.jp/) (Tsunoda and Takagi, 1999).

#### Luciferase reporter assay

pGL3-GPT2 promoter-LUC was co-transfect with an internal control Renilla luciferase expressing CMV-Renilla. Forty-eight hours after transfection, cells were collected for Luciferase assay according to the manufacturer's instructions (Promega). Luminescence was measured with a Lumat LB Single Tube Luminometer (Berthold). Relative luciferase activity was expressed as the ratio of luminescence normalized to the internal control. The luciferase reporter assay was performed in triplicate in at least three separate transfection experiments.

#### Chromatin immuno-precipitation (ChIP) assay

Approximately 2 × 10^6^ C2C12 cells were fixed for 10 min at 37°C by adding 1% formaldehyde to the growth medium. Fixed cells were harvested, and the pellet was resuspended in 1 mL of lysis buffer containing protease inhibitors (200 mM phenylmethylsulfonyl fluoride, 1 μg/mL aprotinin). The lysates were sonicated to obtain DNA fragments of 200–1000 bp. Sonicated samples were centrifuged, and the soluble chromatin was diluted 10-fold in dilution buffer and used directly for ChIP assays. An aliquot (1/100) of sheared chromatin was further treated with proteinase K, extracted with phenol/chloroform and precipitated to determine DNA concentration and shearing efficiency (“Input DNA”). Briefly, the sheared chromatin was pre-cleared for 2h with 1 μg of non-immune IgG (Calbiochem, Burlington, MA, USA) and 30μL of Protein G Plus/Protein A Agarose suspension (Calbiochem) saturated with salmon sperm (1mg/mL). Precleared chromatin was divided in aliquots and incubated at 4°C for 16h with 1 μg of anti-THR antibody (C3) (ab2743, Abcam). After five rounds of washing, bound DNA-protein complexes were eluted by incubation with 1% sodium dodecyl sulfate-0.1M NaHCO_3_ elution buffer. Formaldehyde cross-links were reversed by incubation in 200 mM NaCl at 65°C. Samples were extracted twice with phenol-chloroform and precipitated with ethanol. DNA fragments were recovered by centrifugation, resuspended in 50 μL H_2_O, and used for Real Time PCRs.

#### MTT assay

Cells were plated in 96-well plates at 2.000 cells per well and 24-well plates at 1 X 10^4^ cells per well in complete DMEM (20 mM glucose, 2 mM glutamine, 10% dFBS; Invitrogen). After 24h, cells were washed with PBS, and changed to either glutamine-free DMEM (with 20 mM glucose) or glucose-free DMEM (with 2 mM glutamine) containing 10% dFBS. At different time points cells were stained with 1% crystal violet in 20% ethanol for 10 min at room temperature. Adherent stained cells were visualized under a light microscope at 4X magnification. Cell viability was measured at same time points by the 3-(4,5-Dimethylthiazol-2-yl)-2,5-diphenyltetrazolium bromide (MTT; Sigma Aldrich) assay according to the manufacturer's instructions. Briefly, 20 μL of 5 mg/mL MTT solution in PBS was added to each well and then incubated for 4 h at 37°C, 5%CO2. Cell viability dependent absorbance of the dark blue formazon crystals was measured in a spectrophotometer (wavelength 570nm).

#### GPT2 enzyme activity assay

GPT activity was analyzed using the alanine aminotransferase (ALT) Assay Kit (ab105134, Abcam). Cells were homogenized in ice-cold ALT Assay buffer and centrifuged. GPT activity was detected by measuring OD570 nm.

#### Real time qRT-PCR

Messenger RNAs were extracted with Trizol reagent (Life Technologies). Complementary DNAs were prepared with SuperScript VILO Master Mix (Life Technologies) as indicated by the manufacturer. The cDNAs were amplified by PCR in a CFX Connect Real-Time PCR Detection System (Bio-Rad) with the fluorescent double-stranded DNA-binding dye SYBR Green (BioRad). Specific primers for each gene were designed to work under the same cycling conditions (95°C for 10 min followed by 40 cycles at 95°C for 15 s and 60°C for 1 min), thereby generating products of comparable sizes (about 200 bp for each amplification). Primer combinations were positioned whenever possible to span an exon–exon junction and the RNA digested with DNase to avoid genomic DNA interference. Primer sequences are reported in the Table S2. The relative amounts of gene expression were calculated using Cyclophilin-A as an internal standard and in some qRT-PCR we used the 18S oligonucleotides for internal standard as additional normalizer (Figure S1F). All samples were run in triplicate. The results, expressed as N-fold differences in target gene expression, were determined as follows = 2^-(ΔCt target−ΔCt control)^ (Livak and Schmittgen, 2001).

#### Denervation experiments

12-weeks-old C57BL/6 male mice (The Jackson Laboratory, Bar Harbor, Maine, USA) were divided into 4 groups of 15 mice per group: CTR, TH, α-KG and Gln. TH group included mice made hyperthyroid by adding THs (1 mg/mL T3 + 4 mg/mL T4) in drinking water for 1 week. α-KG was provided with 3% α-KG sodium salt in drinking water for 1 week. Gln group included mice receiving a Gln enriched diet for 1 week. Control group was provided with normal diet *ad libitum* and sterile drinking water. After a week, sciatic nerve cut was conducted as described previously (Bentzinger et al., 2013). Following denervation each group received the respective supplements for another 1 week. At the end of the experiment, the mice were humanely killed to collect blood and muscle tissue samples for further testing. The muscles were stored at −80°C for Real Time quantitative PCR, Western Blot and histological analyses. In order to analyze the role of GPT2 in the TH-dependent protection from denervation-induced muscle atrophy, we also performed denervation experiments in GPT2 KO^+/-^ mice. These mice were subdivided in two groups of 15 mice per group, CTR and TH, subjected to the above-described denervation protocol and compared to wild type mice. Animal experimental protocols were approved by the Animal Research Committee of the University of Naples “Federico II”.

#### Mito tracker staining

Mitochondrial activity was measured using the MitoTracker Red CMXRos dye (M7512, Invitrogen), according to the manufacturer's instructions. Briefly, after 2 days of denervation, hindlimb muscles were dissected, digested into mononuclear cells and incubated in pre-warmed PBS containing 50 nM MitoTracker in the dark at 37°C for 30 min. The cells were washed twice prior to analysis using the FACS Canto2 flow cytometer (Becton Dickinson, USA). In order to analyze the mitochondrial distribution, dissected extensor digitorum longus (EDL) muscle fibers were incubated for 30 min at room temperature with 200nM MitoTracker RedCMXRos. The digital images of MitoTracker fluorescence were acquired with a confocal laser scanning microscope (Leica) equipped with a 63X 1.4 NA water immersion objective. Images were obtained using the Leica Image Browser and processed using the Photoshop CS5 (Adobe) software package. The Mitotracker integrated density was measured using the ImageJ software.

#### GC/MS and isotope-tracing analysis

Breefly, pulse isotopic labeling was performed by feeding C2C12 TET-ON D2 cells at 40–50% of confluence with 25 mM [^13^C6]-glucose in DMEM no Glucose or with 2.5 mM [^15^N]-glutamine or 2.5 mM [^13^C]-glutamine (Cambridge Isotope laboratories) in DMEM no Gln in the absence of FBS and treated with 2 μg/mL DOX and THs (30 nM T3 and 30 nM T4) for 48h. Upon treatment, cells were rinsed three times in PBS to be then homogenized in 1 mL of pre-chilled methanol/water 1:1 solution containing 10 nmol of internal standards. Samples were centrifuged at 10.000 g for 10 min at 4°C. The resulting supernatants were collected and transferred into new eppendorf tubes and dried using a speedvac. Samples were then resuspended in 1 mL of acetonitrile and centrifuged again. Insoluble material was removed by centrifugation at high speed for 10 min at 4°C. Samples were solubilized in pyridine (50μL) and derivatized with 25 μL of N,O-Bis(trimethylsilyl(TMS)trifluoroacetamide (BSTFA) with a reaction time of 90 min. One μL was injected, split ratio 1:10. GC-MS analyses were carried out on a Shimadzu GCMS 2010plus (Kyoto, Japan) with the following parameters. Injection temperature 280°C, Ramp 0–1.00 min 100°C, 1.00–6.00 min 100–320°C, hold for 2.33 min. Column flow 1.10 mL/min Linear velocity 39 cm/s. Helium gas was used. Ion source Temp. 200°C, Interface 320°C, Solvent cut 5.9 min, Scan 35–600 m/z. Detector voltage 0.1 kV. Tentative identification of compounds based on accurate MS measurements and isotopic fine distribution (ISF) was performed by Metaboscape 4.0. Metabolites signals were normalized using internal standards. Comparisons and differences were analyzed for statistical significance by two-way ANOVA test and Bonferroni post-test analysis. All graphs, bars or lines indicate means and standard errors of the mean (s.e.m.).

#### Antibodies

All primary antibodies were used at 1/1000 for Western Blot; when the antibody was used for IHC, the dilution is indicated in the list. The following primary antibodies were used: a rabbit anti–full-length GPT2 antibody (16757-1-AP, 1/100 for IHC) from Proteintech; anti-PKB/Akt (#9272), Phospho-AktSer473 (#9271), p70 S6 kinase (#9202) and Phospho-p70 S6 kinaseThr389 (#9205) from Cell Signaling. Anti-Skeletal Myosin, Slow (M8421) and Skeletal Myosin Fast (M4276) from Sigma Aldrich. Anti-Myosin Heavy Chain types IIA (SC-71; 1/50 for IHC) and IIB (BF-F3; 1/50 for IHC) from the Developmental Studies Hybridoma Bank, Anti-Laminin (ab11575; 1/500 for IHC) from Abcam and anti-GAPDH (E-AB-20059) from Elabscience.

#### Western blot analysis

Total protein extracts from cells and muscles were run on a 10% SDS-PAGE gel and transferred onto an Immobilon-P transfer membrane (Millipore, Burlington, MA, USA). The membrane was then blocked with 5% non-fat dry milk in PBS, probed with primary antibodies overnight at 4°C, washed, and incubated with horseradish peroxidase–conjugated anti-rabbit immunoglobulin G secondary antibody (1:3000), and detected by chemiluminescence (Millipore, cat. WBKLS0500). After extensive washing, the membrane was incubated with anti-Tubulin or anti-GAPDH antibodies as loading control. All Western Blot were run in triplicate, and bands were quantified with ImageJ software.

#### Animals, histology, and immunostaining

Muscles were dissected and frozen in liquid nitrogen-cooled isopentane; 8 μm muscle cryosections were used for histology analyses. Cryostat sections were stained with Hematoxylin/Eosin (HE) or Sirius Red (Sigma-Aldrich, St Louis, MO, USA) according to classical methods (Dubowitz, 1974; Ardite et al., 2012). Briefly, for H&E analysis, cross sections were fixed in 4% formaldehyde at room temperature for 15 min and stained with H&E. Fiber size distribution was quantified by ImageJ software. Up to 6 fields of view were captured from the same location within each muscle. Six hundred myofibers were measured per muscle. For Sirius Red staining, cryosections were sequentially incubated with a solution containing 0.1% Fast green in saturated picric acid, washed with distillated water, incubated with 0.1% Fast green and 0.1% Sirius Red in saturated picric acid, washed with distillated water. Sections were dehydrated by a series of increasing alcohol concentrations sequentially: 50% ethanol and picric acid, 70%, 96%, 100% ethanol and lastly xylene. The images were acquired by Leica DMi8 microscope and quantification of collagen content in muscle was performed according to Ardite et al. (Ardite et al., 2012) using ImageJ software. For MHC staining, sections were fixed with 100% methanol. For GPT2 staining, sections were fixed with PFA 4% followed by microwave oven antigen retrieval in citric acid. Sections were then blocked with 3% normal goat serum and 0.3% Triton X-100 in PBS. They were sequentially incubated with the primary antibody overnight at 4°C and secondary fluorescent antibodies (Invitrogen) for 1 h at room temperature. Then, nuclei were stained with DAPI and sections mounted in 80% Glycerol. Images were captured using the Leica Application Suite LAS X Imaging Software with a fluorescent Leica DMi8 microscope. Methods of SDH staining were described elsewhere (Romanino et al., 2011). Samples were dehydrated and mounted with Eukitt mounting medium (Bio-Optica).

### Quantification and statistical analysis

The results are shown as the mean ± standard deviation (SD) throughout as specified in figure legends. Differences between samples were assessed by the Student s two-tailed t test for independent samples. Results of GC/MS analysis were corrected by using the two-way ANOVA test and Bonferroni post-test analysis. Relative mRNA levels (in which the control sample was arbitrarily set as 1) are reported as results of Real Time qRT-PCR, in which the expression of Cyclophilin-A served as housekeeping gene. In all experiments, differences were considered significant when p value (P) was less than 0.05. Asterisks indicate significance at ^∗^p < 0.05, ^∗∗^p < 0.01, and ^∗∗∗^p < 0.001 throughout. Definitions of n-values are reported in figure legends.

## Data Availability

All data reported in this paper will be shared by the lead contact upon request. Original Western blot images have been deposited at Mendeley and are publicly available as of the date of publication. The DOI is listed in the key resources table. This paper does not report original code. Any additional information required to reanalyze the data reported in this paper is available from the lead contact upon request.

## Supplemental information

Document S1. Figures S1–S7 and Tables S1 and S3

Table S2. Oligonucleotides used in this study, related to STAR Methods

Document S2. Article plus supplemental information
