# Thyroid hormone regulates glutamine metabolism and anaplerotic fluxes by inducing mitochondrial glutamate aminotransferase GPT2

**DOI:** 10.1016/j.celrep.2022.110562

**Published:** 2022-03-22

**Authors:** Annunziata Gaetana Cicatiello, Serena Sagliocchi, Annarita Nappi, Emery Di Cicco, Caterina Miro, Melania Murolo, Mariano Stornaiuolo, Monica Dentice

## Main text

(Cell Reports *38*, 110409, February 22, 2022)

Due to an oversight, in the originally published version of this article, the graphical abstract contained an error in which the arrow between GLN and GLU was in the wrong direction. The corrected graphical abstract now appears with the article online, and the original and corrected graphical abstract appear here.

Cell Press and the authors regret this error.

**Figure:**
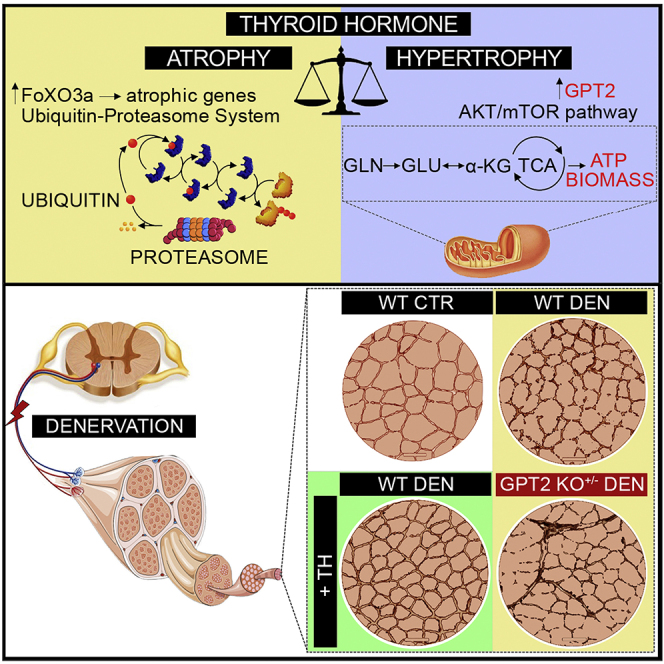
(Corrected)

**Figure:**
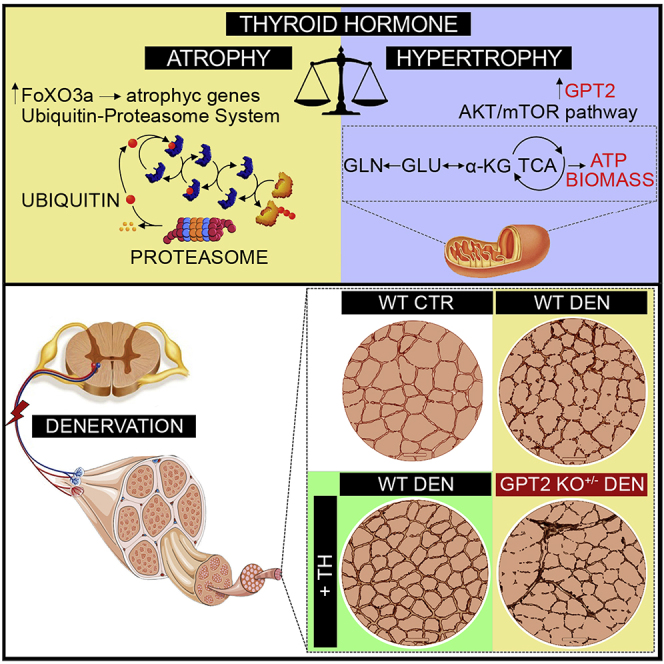
(Original)

