# Supplementary material for: Thyroid hormone regulates glutamine metabolism and anaplerotic fluxes by inducing mitochondrial glutamate aminotransferase GPT2
Source: Cell Rep. 2022 Feb 22;38(8):110409. doi: 10.1016/j.celrep.2022.110409 (PMC8889437; doi:10.1016/j.celrep.2022.110409)
Supplement: Document S1. Figures S1–S7 and Tables S1 and S3 [file mmc1.pdf]

Cell Reports, Volume *38*

Supplemental information

Thyroid hormone regulates glutamine metabolism and anaplerotic fluxes by inducing mitochondrial glutamate aminotransferase GPT2

Annunziata Gaetana Cicatiello, Serena Sagliocchi, Annarita Nappi, Emery Di Cicco, Caterina Miro, Melania Murolo, Mariano Stornaiuolo, and Monica Dentice

Supplemental Informations - Supplemental Figures

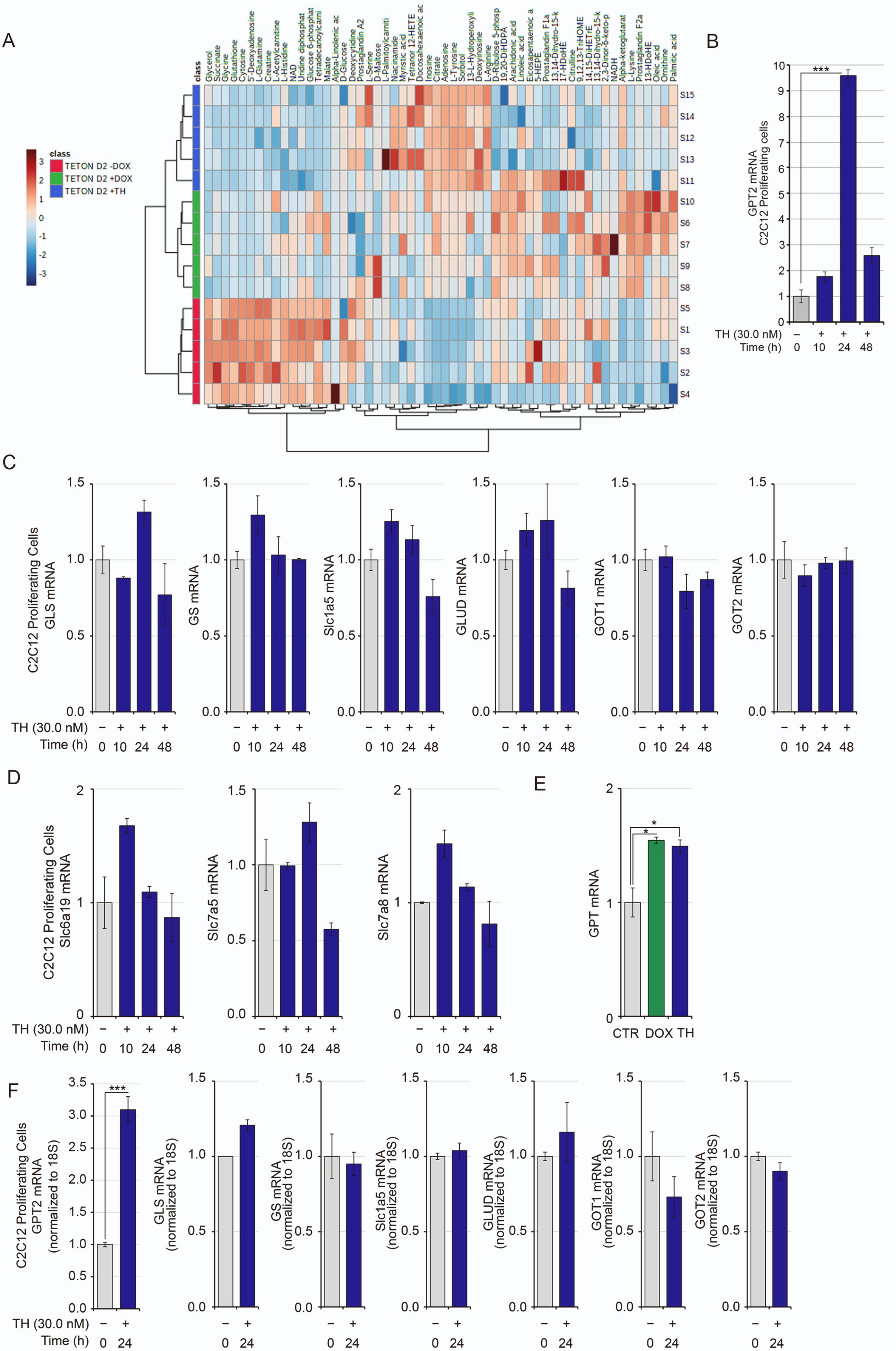

Figure S1

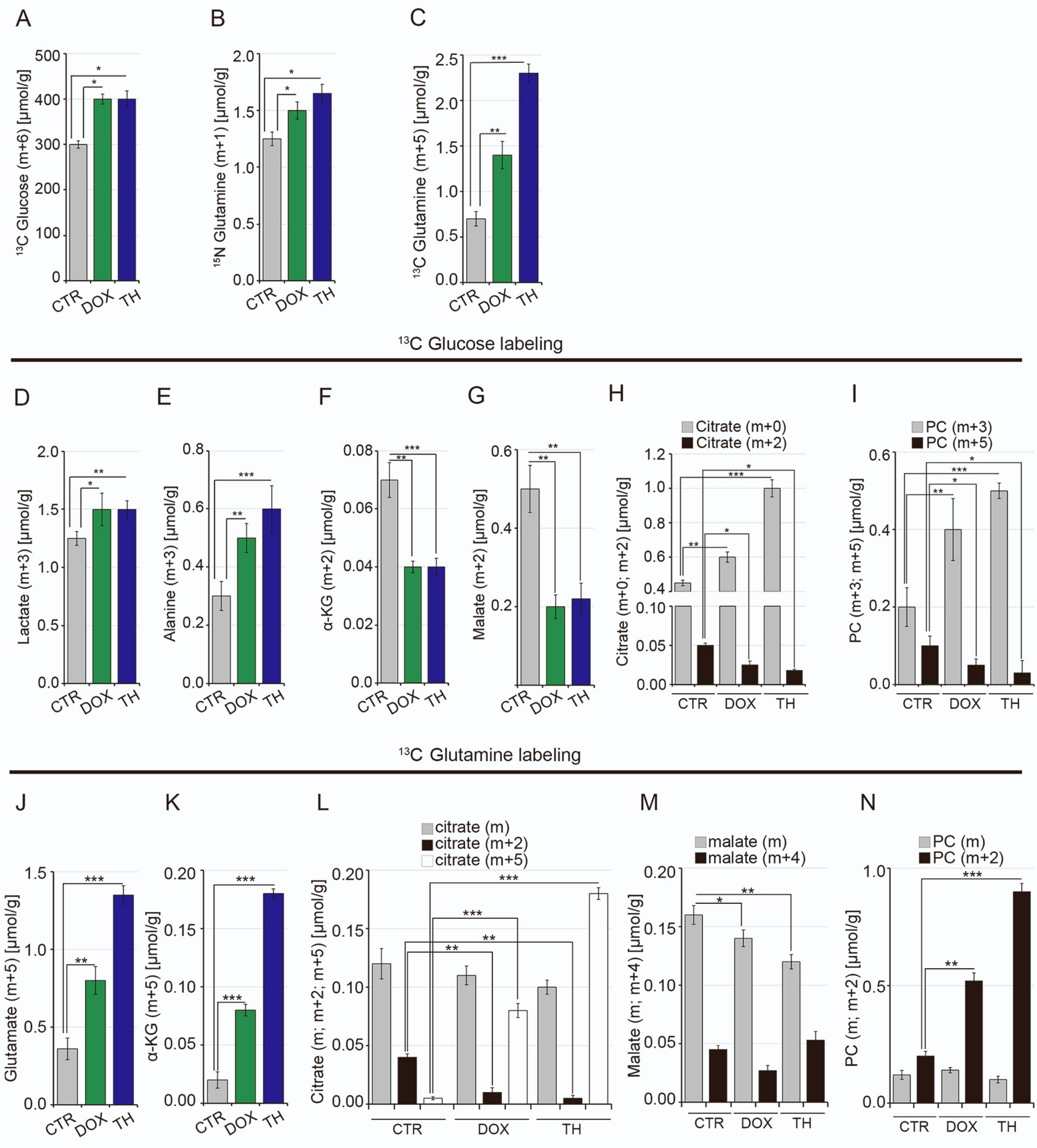
A
B
C
¹³C Glucose (m+6) [μmol/g]
¹⁵N Glutamine (m+1) [μmol/g]
¹³C Glutamine (m+5) [μmol/g]
CTR DOX TH
¹³C Glucose labeling
D
E
F
G
H
I
Lactate (m+3) [μmol/g]
Alanine (m+3) [μmol/g]
α-KG (m+2) [μmol/g]
Malate (m+2) [μmol/g]
Citrate (m+0)
Citrate (m+2)
Citrate (m+0; m+2) [μmol/g]
PC (m+3)
PC (m+5)
PC (m+3; m+5) [μmol/g]
¹³C Glutamine labeling
J
K
L
M
N
Glutamate (m+5) [μmol/g]
α-KG (m+5) [μmol/g]
citrate (m)
citrate (m+2)
citrate (m+5)
Citrate (m; m+2; m+5) [μmol/g]
malate (m)
malate (m+4)
Malate (m; m+4) [μmol/g]
PC (m)
PC (m+2)
PC (m; m+2) [μmol/g]

Figure S2

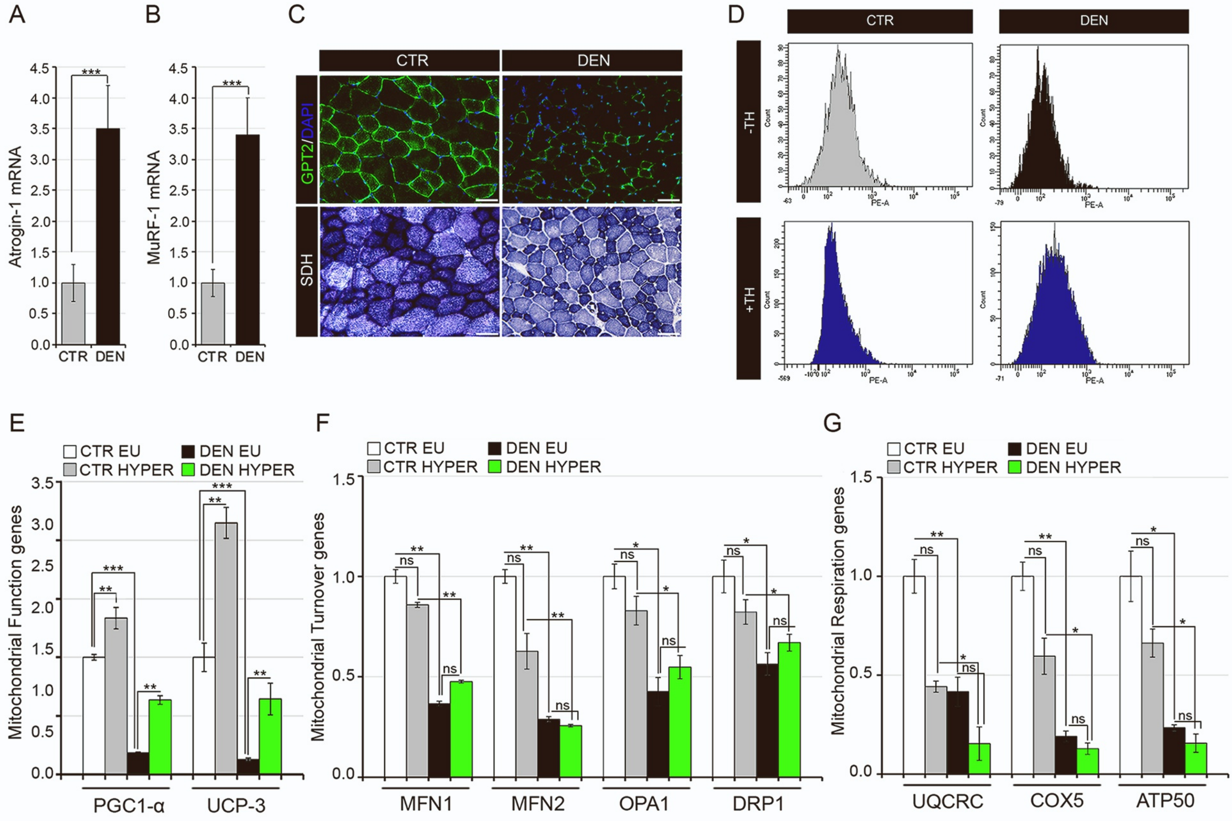
A
B
C
D
Atrogin-1 mRNA
MuRF-1 mRNA
CTR
DEN
GPT2/DAPI
SDH
-TH
+TH
Count
PE-A
E
F
G
CTR EU
DEN EU
CTR HYPER
DEN HYPER
Mitochondrial Function genes
Mitochondrial Turnover genes
Mitochondrial Respiration genes
PGC1-α
UCP-3
MFN1
MFN2
OPA1
DRP1
UQCRC
COX5
ATP50

Figure S3

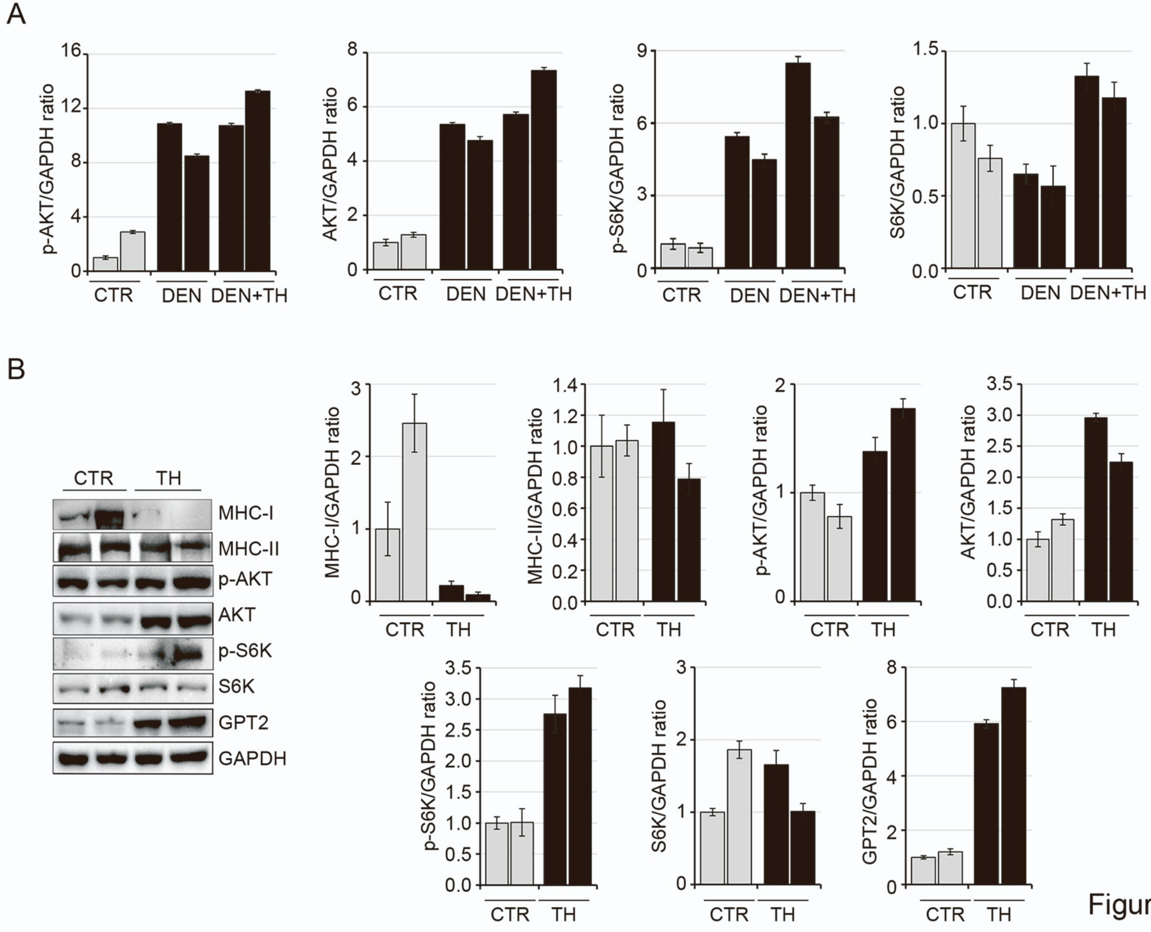

A
p-AKT/GAPDH ratio
AKT/GAPDH ratio
p-S6K/GAPDH ratio
S6K/GAPDH ratio
CTR
DEN
DEN+TH
B
CTR
TH
MHC-I
MHC-II
p-AKT
AKT
p-S6K
S6K
GPT2
GAPDH
MHC-I/GAPDH ratio
MHC-II/GAPDH ratio
GPT2/GAPDH ratio

Figure S4

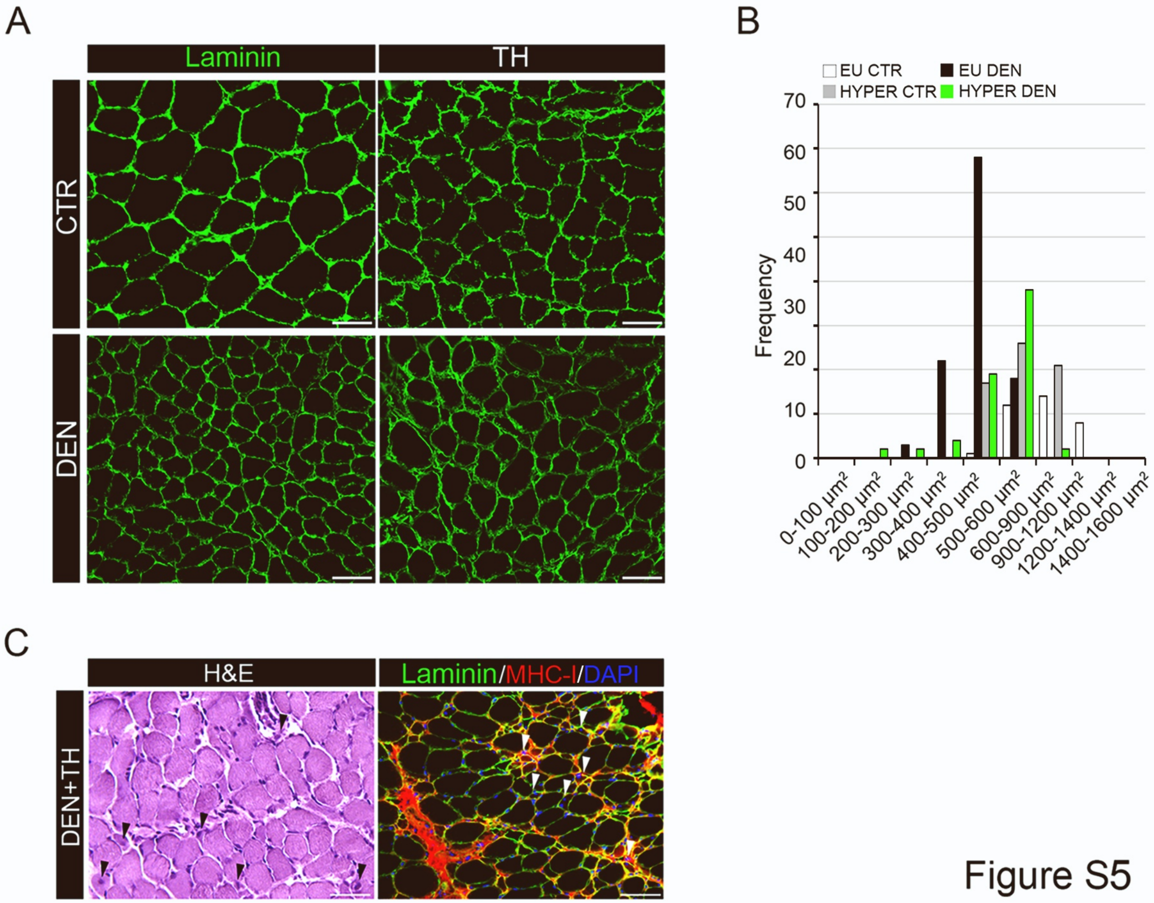
A
Laminin
TH
CTR
DEN
B
EU CTR
EU DEN
HYPER CTR
HYPER DEN
Frequency
0
10
20
30
40
50
60
70
0-100 μm²
100-200 μm²
200-300 μm²
300-400 μm²
400-500 μm²
500-600 μm²
600-900 μm²
900-1200 μm²
1200-1400 μm²
1400-1600 μm²
C
H&E
Laminin/MHC-I/DAPI
DEN+TH

Figure S5

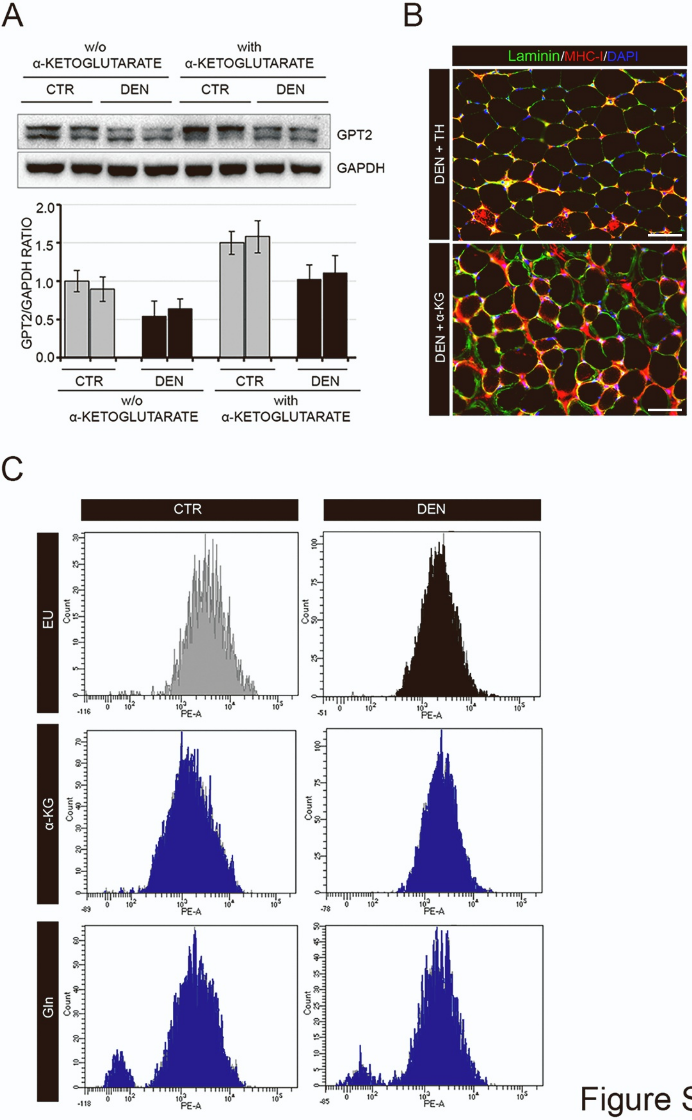
A
w/o
α-KETOGLUTARATE
with
α-KETOGLUTARATE
CTR
DEN
CTR
DEN
GPT2
GAPDH
GPT2/GAPDH RATIO
2.0
1.5
1.0
0.5
0.0
B
Laminin/MHC-I/DAPI
DEN + TH
DEN + α-KG
C
CTR
DEN
EU
α-KG
Gln
Count
PE-A

Figure S6

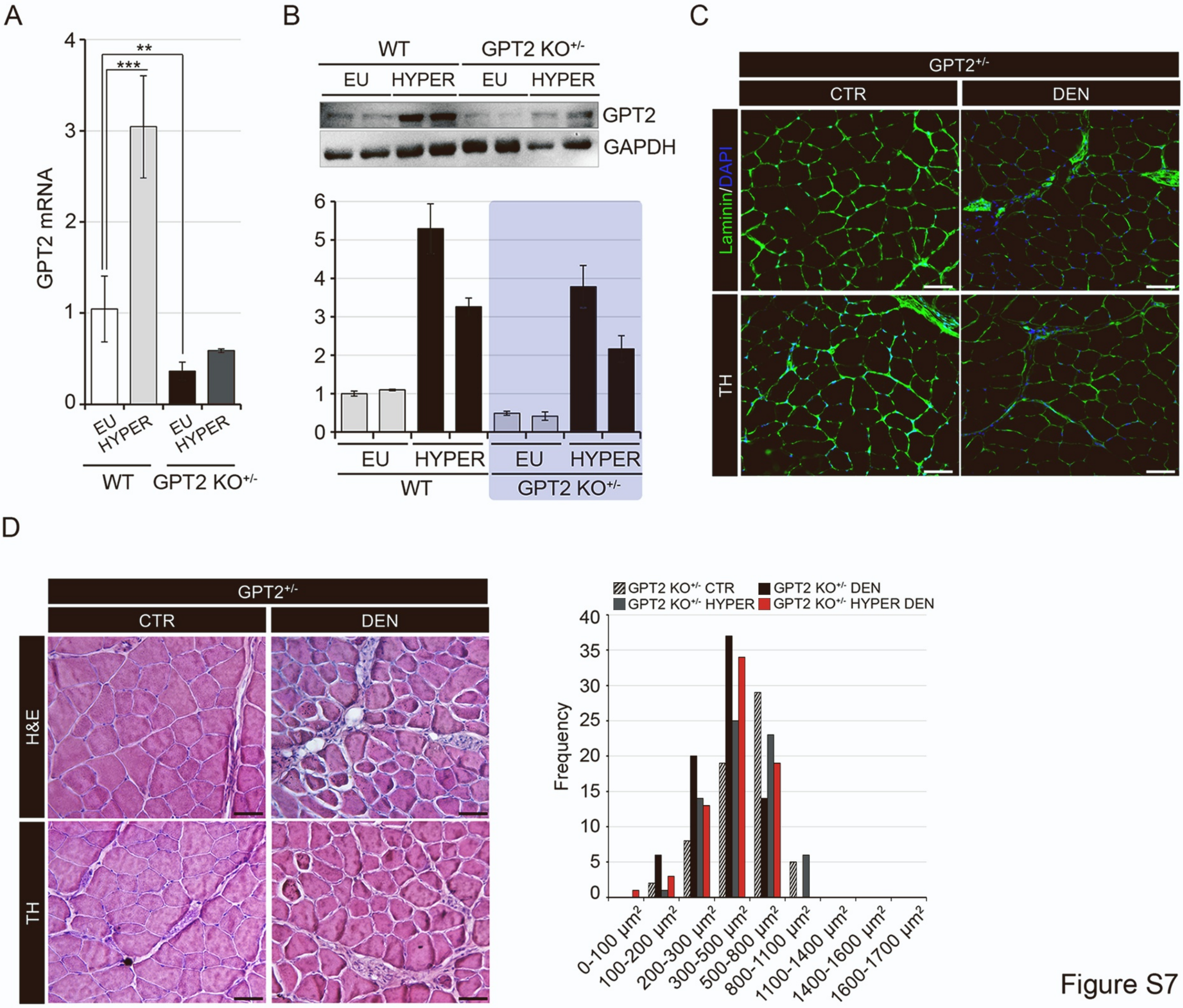
A
GPT2 mRNA
**
***
EU
HYPER
EU
HYPER
WT
GPT2 KO+/-
B
WT
GPT2 KO+/-
EU
HYPER
EU
HYPER
GPT2
GAPDH
EU
HYPER
EU
HYPER
WT
GPT2 KO+/-
C
GPT2+/-
CTR
DEN
Laminin/DAPI
TH
D
GPT2+/-
CTR
DEN
H&E
TH
GPT2 KO+/- CTR
GPT2 KO+/- DEN
GPT2 KO+/- HYPER
GPT2 KO+/- HYPER DEN
Frequency
0-100 μm²
100-200 μm²
200-300 μm²
300-500 μm²
500-800 μm²
800-1100 μm²
1100-1400 μm²
1400-1600 μm²
1600-1700 μm²

Figure S7
